# Proteome analysis of *Aspergillus flavus* isolate-specific responses to oxidative stress in relationship to aflatoxin production capability

**DOI:** 10.1038/s41598-018-21653-x

**Published:** 2018-02-21

**Authors:** Jake C. Fountain, Jin Koh, Liming Yang, Manish K. Pandey, Spurthi N. Nayak, Prasad Bajaj, Wei-Jian Zhuang, Zhi-Yuan Chen, Robert C. Kemerait, R. Dewey Lee, Sixue Chen, Rajeev K. Varshney, Baozhu Guo

**Affiliations:** 1 Department of Plant Pathology, University of Georgia, Tifton, GA USA; 2 USDA-ARS Crop Protection and Management Research Unit, Tifton, GA USA; 3 Center of Excellence in Genomics & Systems Biology, International Crop Research Institute for the Semi-Arid Tropics (ICRISAT), Patancheru, Telangana India; 4 Department of Biology, Genetics Institute, Interdisciplinary Center for Biotechnology Research, University of Florida, Gainesville, FL USA; 5 College of Biology and Environmental Science, Nanjing Forestry University, Nanjing, China; 6 College of Plant Protection, Fujian Agriculture and Forestry University, Fuzhou, Fujian China; 7 Department of Plant Pathology and Crop Physiology, Louisiana State University, Baton Rouge, LA USA; 8 Department of Crop and Soil Sciences, University of Georgia, Tifton, GA USA

## Abstract

*Aspergillus flavus* is an opportunistic pathogen of plants such as maize and peanut under conducive conditions such as drought stress resulting in significant aflatoxin production. Drought-associated oxidative stress also exacerbates aflatoxin production by *A. flavus*. The objectives of this study were to use proteomics to provide insights into the pathogen responses to H_2_O_2_-derived oxidative stress, and to identify potential biomarkers and targets for host resistance breeding. Three isolates, AF13, NRRL3357, and K54A with high, moderate, and no aflatoxin production, were cultured in medium supplemented with varying levels of H_2_O_2_, and examined using an iTRAQ (Isobaric Tags for Relative and Absolute Quantification) approach. Overall, 1,173 proteins were identified and 220 were differentially expressed (DEPs). Observed DEPs encompassed metabolic pathways including antioxidants, carbohydrates, pathogenicity, and secondary metabolism. Increased lytic enzyme, secondary metabolite, and developmental pathway expression in AF13 was correlated with oxidative stress tolerance, likely assisting in plant infection and microbial competition. Elevated expression of energy and cellular component production in NRRL3357 and K54A implies a focus on oxidative damage remediation. These trends explain isolate-to-isolate variation in oxidative stress tolerance and provide insights into mechanisms relevant to host plant interactions under drought stress allowing for more targeted efforts in host resistance research.

## Introduction

*Aspergillus flavus* (Link ex Fr, Teleomorph: *Petromyces flavus*) is a facultative plant pathogen, which is capable of infecting maize and peanut. The infection of these crops by *A. flavus* poses a serious threat to human and animal health due to its production of carcinogenic mycotoxins, termed as aflatoxins, resulting in contamination of foodstuffs and livestock feed^1–5^. Aflatoxin contamination also leads to significant economic losses globally due to lost crop value and regulatory restrictions on import and export of contaminated materials^6^.

Research efforts have been focused on prevention of both pre-and post-harvest aflatoxin contamination^7,8^. Pre-harvest aflatoxin contamination is managed mainly through host genetic resistance, irrigation, insect control, and the application of atoxigenic biological control isolates of *A. flavus*, such as Aflaguard (NRRL21882) and AF36 (NRRL18543) which compete with toxigenic isolates for available niches in the environment^9–13^. Host resistance to *A. flavus* colonization and aflatoxin contamination is highly quantitative rather than specific gene-for-gene resistance^14,15^. This resistance is also highly influenced by abiotic stresses such as drought and heat stress which have been shown to significantly exacerbate aflatoxin contamination^16^. Given that drought stress is one of the primary predisposing factors contributing to aflatoxin contamination, understanding the interaction between host plants and *A. flavus* and other aflatoxigenic species is important for developing novel avenues of enhancing host resistance.

Drought stress has been shown to stimulate the production of reactive oxygen species (ROS) in plant tissues which function in stress-responsive signaling and in the initiation of pathogen defense signaling, but can also have deleterious effects on hosts if they accumulate in excessive concentrations^17,18^. Recent studies have suggested that these ROS and related signaling compounds produced by plants such as oxylipins may influence the production of aflatoxin by *A. flavus* during colonization^19^. Previous reports have shown that oxidative stress can stimulate the production of aflatoxin by *A. flavus* with medium amendment with oxylipins, ROS, or ROS production inducers resulting in greater aflatoxin production^20–23^. Conversely, supplementation of medium with ROS scavengers and antioxidant compounds have been shown to reduce or inhibit aflatoxin production^24^. These results suggest that oxidative stress may indeed be a pre-requisite for aflatoxin production^22^. Although the biochemical processes involved in the biosynthesis of aflatoxin have been well characterized^25–27^, there is limited understanding on the regulating mechanisms wherein several transcription factors have been found to be involved. For example, AflR is a key regulatory transcription factor for aflatoxin production whose silencing impedes aflatoxin production^28^. Other transcription factors such as the bZIP transcription factors AtfA and AtfB also bind to aflatoxin gene promoters and regulate oxidative stress responses^29^.

Given the evident relationship between oxidative stress and aflatoxin production in *A. flavus* and related *Aspergillus spp*., and the potential role of oxidative stress and ROS in communication between this pathogen and its hosts under drought stress, investigating the influence of oxidative stress on *A. flavus* may provide insights into the cause of exacerbated aflatoxin contamination under drought and novel means of preventing it. To begin investigating this possibility, we previously examined the transcriptomes of several field isolates of *A. flavus* to oxidative stress when utilizing both aflatoxin conducive or non-conducive substrates^30,31^. In these studies, isolates producing higher levels of aflatoxin and possessing greater tolerance to oxidative stress exhibited less differential gene expression compared to less tolerant, atoxigenic isolates. Interestingly, the pathways regulated in these isolates indicated a possible role for secondary metabolites such as aflatoxin, kojic acid, and aflatrem in oxidative stress responses, along with carbohydrate metabolic pathways, antioxidant mechanisms, and fungal developmental genes. However, few genes were differentially expressed in the highly tolerant isolates indicating a potential role for post-transcriptional and protein-level regulation in oxidative stress tolerance at the examined time point. The role of protein-level interactions in the regulation of aflatoxin production and fungal developmental and reproductive processes have been well demonstrated. For example, G-protein and protein kinase A (PkaA) signaling pathways are used to phosphorylate AflR to regulate aflatoxin production along with conidia and sclerotia development^32,33^. Post-transcriptional modifications have also been suggested based on the low correlation between their RNA sequencing gene expression levels and observed fold-changes in proteins, such as in the case of *A. flavus* responses to temperature stress^34^.

Given the possibility of protein-level regulation, and the fact that protein enzymatic activities are likely to be more directly responsible for the observed variation in isolate-to-isolate variation in oxidative stress responses, here we examined the proteomic responses of select field isolates of *A. flavus* to oxidative stress in aflatoxin production conductive medium using isobaric tags for relative and absolute quantitation (iTRAQ) proteomics. Correlative analyses between these proteomic data and previously obtained transcriptomic data were also performed to examine for possible post-transcriptional regulation of responses. The selected isolates exhibited distinct responses to oxidative stress with the highly stress tolerant and aflatoxigenic isolate AF13 showing more differential expression of developmental, pathogenicity, and secondary metabolite proteins which likely contribute to increased stress tolerance, host infective capability, and competitive advantage with other soil microbes compared to the less toxigenic isolate NRRL3357 and the atoxigenic isolate K54A. The latter two isolates showed more differential expression of carbohydrate metabolic proteins and repair-related proteins indicating a greater focus on oxidative damage remediation possibly due to less effective means of detoxifying ROS. These identified pathways provide insights into important mechanisms related to host interactions, and potential targets for enhancing host resistance using biotechnology.

## Results

### Proteome profiling of *A. flavus* isolate responses to oxidative stress

**Figure 1:**
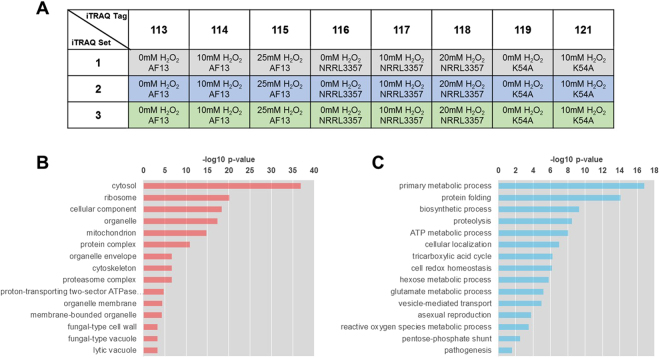
Comparative iTRAQ proteomics analysis. (**A**) Design of the iTRAQ proteomics analysis. Column headings describe the isobaric tag molecular weights utilized for each sample. The rows represent the independent replicated runs of the experiment which are considered as three biological replicates. (**B**) Subcellular localization enrichment for the 1,173 proteins detected in at least two biological replicates. (**C**) Select functional enrichment analysis for gene ontology (GO) biological functions related to oxidative stress responses.

In order to examine the differences in the responses of highly toxigenic, moderately toxigenic, and atoxigenic isolates of *A. flavus* to oxidative stress as encountered in the field during drought stress and the colonization of stressed host plant tissues, a comparative proteomic analysis was performed on select isolates following treatment with various levels of H_2_O_2_. H_2_O_2_ concentrations of 0 mM, 10 mM, and 20/25 mM representing low, moderate, and high levels of oxidative stress, respectively, were used and based on previous observations of fungal growth and stress responses at these concentrations^15,21,30^. We used an iTRAQ approach to identify proteins differentially expressed in response to stress. This study was performed with three biological replicates, each as an independent iTRAQ set (Fig. 1A). Approximately 360,000 MS/MS spectra were generated for each biological replicate. Following filtration using a global false discovery rate (FDR) of 1%, 280,365 MS spectra were obtained (Table S1). For each replicate an average of 18,364 distinct peptides were identified using the ProteinPilot software coupled with both the NCBI and Uniprot databases, resulted in the identification of an average of 1,900 proteins in each replicate at a 1% global FDR. Of these 1,900 proteins, 1,173 (61.74%) were present in at least two biological replicates, and, of those, 799 (68.12%) were expressed in all three biological replicates. These 1,173 proteins were then further examined in subsequent analyses.

Gene ontology (GO) enrichment analyses were then performed to provide an overview of cell components and pathways examined by the experiment. Examination of subcellular localization enrichment for the detected proteins showed that cytosolic, ribosomal, and organelle-associated proteins were enriched (Fig. 1B). Biological functional annotation of the detected proteins was also consistent with the localization analysis with primary metabolism, protein metabolism, redox homeostasis, and asexual reproductive process being among the most enriched stress-related GO terms observed (Fig. 1C). Functional and localization GO enrichment in all 1,173 proteins and the 799 proteins expressed in all three biological replicates showed highly similar results (Figure S1)

### Differential expression analysis of the detected proteins

**Figure 2:**
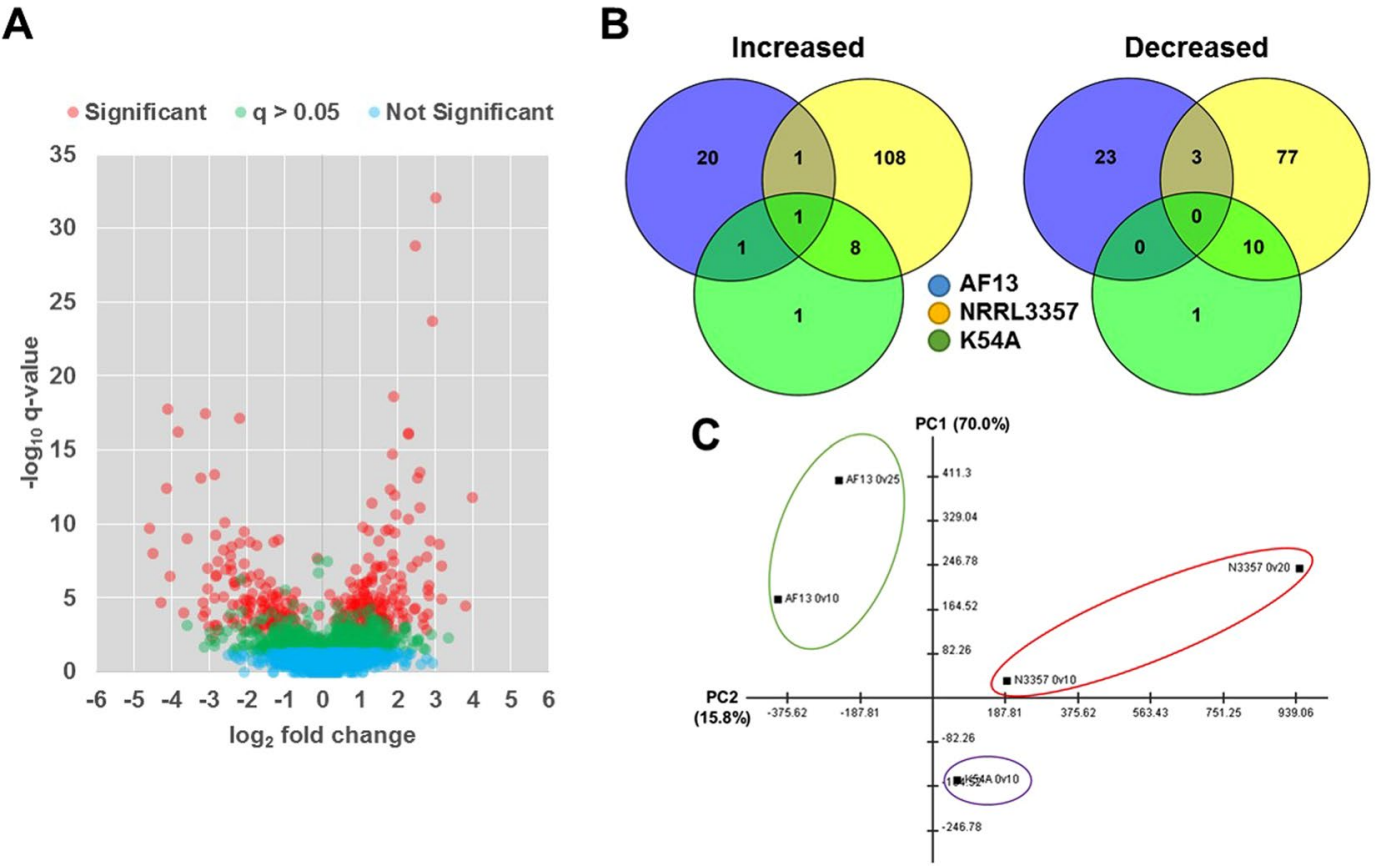
Differential expression analysis. (**A**) Volcano plot of detected proteins indicating significantly (red), non-significantly with q-values > 0.05 (green), and non-significantly (blue) differentially expressed proteins. (**B**) Venn diagrams of proteins increased or decreased in expression in response to increasing stress levels in AF13 (blue), NRRL3357 (yellow), and K54A (green). (**C**) Principal components analysis of the protein expression profiles detected for each isolate. Isolate groups are delineated with the colored circles.

Proteins were considered to be differentially expressed if they exhibited a fold change ≥1.2 or ≤0.8 with a *p*-value ≤ 0.05 in each biological replicate and with a *q*-value ≤ 0.05 (Fig. 2A). The AF13 isolate, which was previously found to tolerate higher levels of oxidative stress and produce high levels of aflatoxin^21^, exhibited 40 and 14 differentially expressed proteins (DEPs) under 10 mM and 25 mM H_2_O_2_ treatments, respectively, compared to 0 mM H_2_O_2_ control (Table 1). The NRRL3357 isolate, which tolerates moderate levels of oxidative stress and produces moderately high levels of aflatoxin^21^, exhibited 27 and 202 DEPs under the 10 mM and 20 mM H_2_O_2_ treatments, respectively (Table 1). Lastly, the K54A isolate, which previously tolerated the least amount of oxidative stress of isolates surveyed and is atoxigenic^21^, exhibited 22 DEPs comparing between the control and the 10 mM H_2_O_2_ treatment (Table 1). A list of the DEPs found within each isolate and treatment can be found in Table S2.

**Table 1:** Numbers of significantly, differentially expressed proteins.

Isolate	Toxin^a^	H_2_O_2_ (mM)^a^	0 v 10 mM	0 v 20/25 mM
AF13	+++	35	40	14
NRRL3357	+	20	27	202
K54A	—	15	22	

^a^Aflatoxin production capability (+++, high; +, moderately high; −, atoxigenic) and maximum [H_2_O_2_] tolerance observed in Fountain *et al*.^21^.

Comparison of the DEPs across isolates showed that each exhibited highly distinct responses to oxidative stress with few commonly regulated DEPs being detected between them (Fig. 2B). These distinctive responses can also be seen in the principal components analysis (PCA) which showed clear differences in the overall expression profiles for the isolates in all treatments (Fig. 2C). Of the isolates, the NRRL3357 and K54A isolates exhibited more closely related responses to stress with numerically more commonly regulated proteins and closer relationships indicated in the PCA (Fig. 2B,C). These distinct responses can also be observed in the hierarchical clustering analysis of protein expression between isolates (Figure S2). Here, a clear segregation of expression patterns can be observed, such as when comparing the responses of AF13 and NRRL3357. For example, NRRL3357 exhibited greater levels of variation in protein expression comparing the control and 20 mM H_2_O_2_ treatment while having more commonality with K54A in the 10 mM H_2_O_2_ treatment.

### Functional classification of the differentially expressed proteins

**Figure 3:**
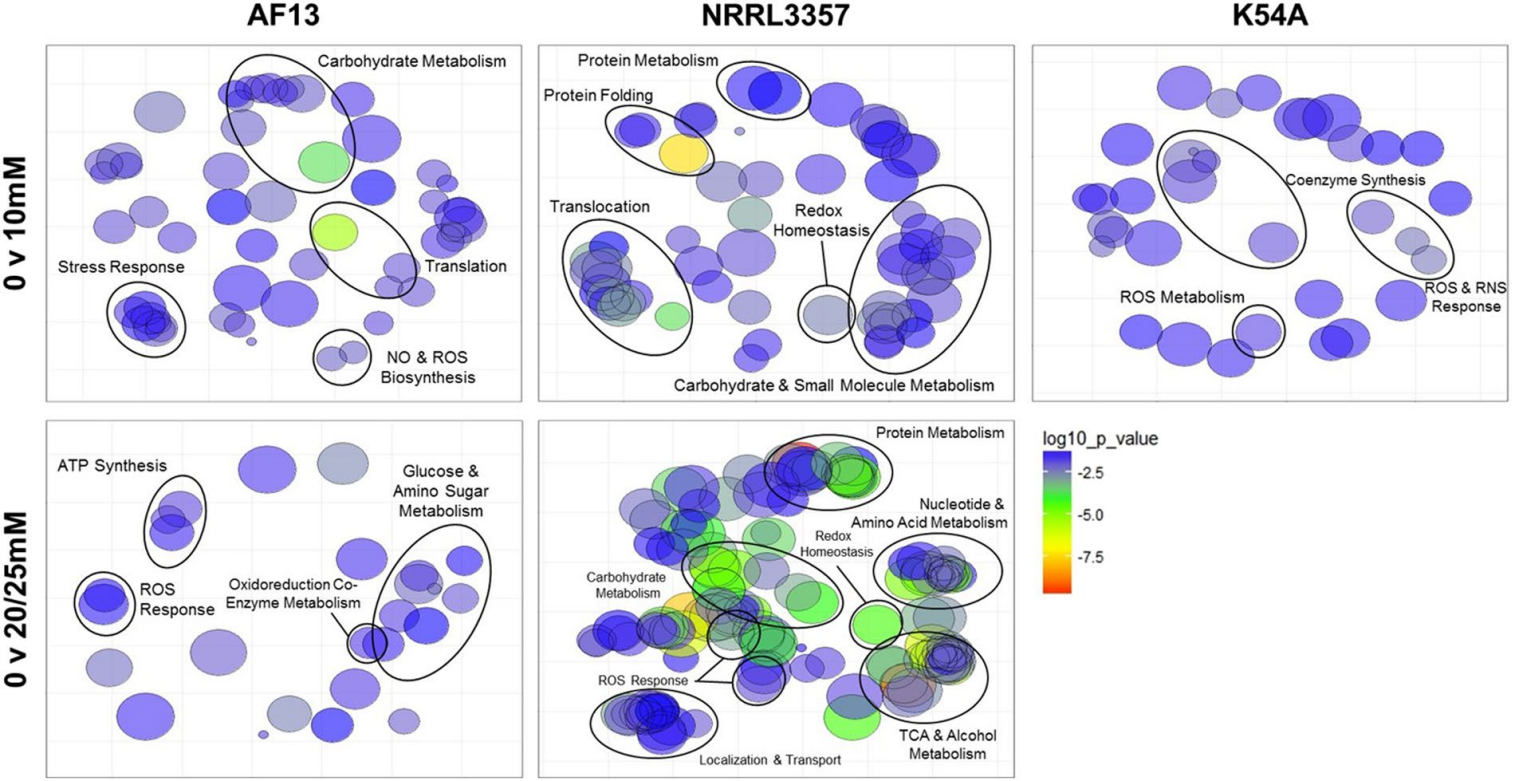
Gene ontology (GO) enrichment for protein biological functions under increasing levels of oxidative stress. Biological function GO term enrichment analysis was performed using REVIGO for DEPs obtained from each isolate and treatment. The size of each GO term is indicative of the relative number of DEPs corresponding to each term. The color of each GO term corresponds to the log_10_ *p*-values obtained for each GO term enrichment which are indicated on the scale.

The DEPs identified in response to increasing oxidative stress in each isolate were used for functional enrichment analyses based on biological process GO. The functional classification of the DEPs was done using FungiDB^35^, and redundant GO terms were removed using REVIGO^36^. The detected functional annotations for each isolate were consistent with oxidative stress responses. AF13 showed enrichment for terms including carbohydrate and tricarboxylic acid cycle components, responses to oxidative stress, protein folding and metabolism, ATP biosynthesis, and nitric oxide (NO) biosynthesis with increasing levels of H_2_O_2_ stress (Fig. 3). NRRL3357 showed a greater variety of enriched terms than observed AF13. This isolate’s terms included those found in AF13 and others such as co-enzyme metabolism, NADPH metabolism, and pyrimidine metabolism under increasing levels of stress (Fig. 3). The DEPs observed in K54A in response to stress included several proteins lacking functional annotations and were, therefore, lacking in GO enrichment compared to the other isolates. The terms found in K54A included those involved in responses to oxidative stress, coenzyme (NADH) biosynthesis, and ROS and RNS metabolism (Fig. 3). A complete list of the enriched GO terms for biological processes found in the isolates can be found in Table S3.

### Sub-cellular localization of the differentially expressed proteins

In addition to biological processes, the sub-cellular localization GO terms for the detected DEPs were examined for enrichment in response to stress. Consistent with the localization analysis described for all 1,173 detected proteins, the DEPs tended to localize to the cytoplasm and membranes including the plasma and organelle membranes. A complete list of the enriched GO terms for cellular localization found in the isolates can be found in Table S4.

For AF13, DEPs under moderate stress tended to localize in the cytoplasm, cell wall, cellular and mating projections, proteasome complexes, and telomeres. Conversely, under higher levels of stress, AF13 DEPs localized more to cytoplasmic and mitochondrial components, particularly to cytochrome complexes and electron transport complexes involved in ATP biosynthesis. For NRRL3357, DEPs under moderate stress localized to the cytoplasm and to membrane-associated components including vesicular transport components, the endoplasmic reticulum, and the Golgi apparatus. Under higher levels of stress, other additional enriched locations including mitochondrial ATP synthase complexes and TCA cycle components, and ribosomes were also detected for the DEPs. For K54A, similar to NRRL3357, DEPs detected under stress tended to localize the cytoplasm, vesicular transport components, ribosomes, the Golgi apparatus, and the mitochondria. Overall for all isolates examined, it is clear that cytoplasm, mitochondria, and vesicle localized responses comprise the bulk of the responses of these isolates to H_2_O_2_.

### KEGG pathway analysis of the differentially expressed proteins

**Figure 4:**
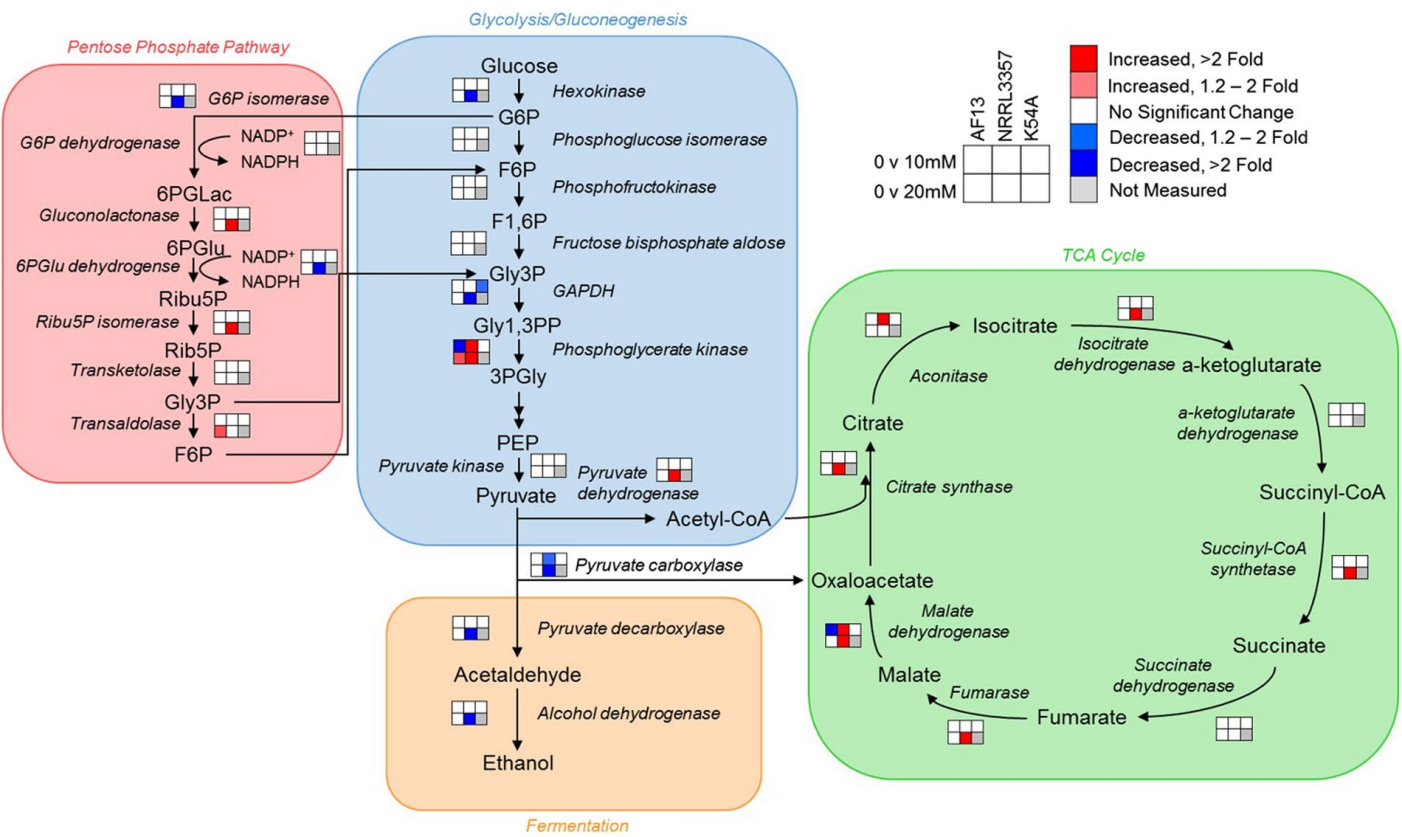
Carbohydrate metabolic pathway components differentially expressed in response to increasing oxidative stress. Enzymes found to be differentially expressed in the glycolysis/gluconeogenesis (blue), pentose phosphate (red), fermentation (yellow), and tricarboxylic acid (TCA) cycle (green) pathways are plotted based on their associations found in the KEGG database. Larger fonts correspond to compounds in the pathways while smaller, italicized fonts represent enzymes. The 2 × 3 heatmaps represent each isolate and fold change in expression observed relative to the control for each H_2_O_2_ treatment. Red and blue colors represent significantly higher and lower expression, respectively; white color represents no significant change in expression; and gray color represents treatments not measured in this experiment. Abbreviations: G6P, glucose-6-phosphate; F6P, fructose-6-phosphate; F1,6 P, fructose-1,6-bisphosphate; Gly3P, glyceraldehyde-3-phosphate; Gly1,3PP, glyceraldehyde-1,3-bisphosphate; PEP, phosphoenolpyruvate; 6PGLac, 6-phosphogluconolactone; 6PGlu, 6-phosphogluconate; Ribu5P, ribulose-5-phosphate; Rib5P, ribose-5-phosphate.

In addition to GO annotation, the pathway annotations for each DEP based on the Kyoto Encyclopedia of Genes and Genomes (KEGG) database were also examined^37^ followed by enrichment analysis. For AF13, under moderate levels of stress, several carbohydrate metabolism-related pathways were identified including starch and sucrose metabolism, glycolysis, and pyruvate metabolism (Fig. 4). In addition, carbon fixation and N-glycan biosynthesis pathways were found to be represented by the DEPs. Under high stress, AF13 DEPs were enriched for pathways including glycolysis, amino and nucleotide sugar metabolism, and oxidative phosphorylation. For NRRL3357 under moderate stress, enriched pathways included carbohydrate metabolism components such as glycolysis, pyruvate metabolism and the TCA cycle (Fig. 4). Amino acid metabolism, benzoate degradation, and fatty acid degradation pathways were also enriched. Under high stress, in addition to those observed under moderate stress, pathways such as the pentose phosphate pathway, N-glycan biosynthesis, and glutathione metabolism were enriched. One component of the aflatoxin biosynthetic pathway, versicolorin A dehydrogenase/ketoreductase (ver-1) was increased (Table S2). While other aflatoxin biosynthesis proteins including polyketide synthase A (pksA), versicolorin B synthase (vbs), and dimethylsterigmatosystin 6-O-methyltransferase (omtB) were detected in the analysis, only ver-1 was found to be differentially expressed in NRRL3357 under high stress conditions. Conversely, one cyclopiazonic acid (CPA) biosynthetic component, a dimethylallyl tryptophan synthase (dmaT; AFLA_139480) was decreased in NRRL3357 but increased in AF13 (Table S2). For K54A, under stress the pathways enriched included arginine and proline metabolism, limonene and pinene degradation, glycolysis (Fig. 4), and oxidative phosphorylation. Overall, for all of the isolates respiration-related pathways such as glycolysis, the TCA cycle, and oxidative phosphorylation along with amino acid and complex macromolecular catabolism comprise a bulk of the pathways differentially regulated when treated with H_2_O_2_ (Table 2). A complete list of the enriched KEGG biological pathways found in the isolates can be found in Table S5.

**Table 2:** Significantly enriched KEGG pathways in all isolates and treatments, and their correlation with transcriptome data.

ID	Annotation	Results	P-value	Benjamini	Bonferroni	r^1^
ec00480	Glutathione metabolism	20	5.72E-05	0.0002	0.0014	0.6797
ec00030	Pentose phosphate pathway	13	0.0004	0.0008	0.0098	0.1893
ec00062	Fatty acid elongation	6	0.0490	0.0490	1.0000	0.1453
ec00281	Geraniol degradation	10	0.0069	0.0098	0.1668	0.0958
ec00640	Propanoate metabolism	31	0.0371	0.0405	0.8904	0.0277
ec00410	beta-Alanine metabolism	12	0.0003	0.0007	0.0081	0.0051
ec00290	Valine, leucine and isoleucine biosynthesis	10	0.0007	0.0013	0.0170	0.0019
ec00620	Pyruvate metabolism	34	3.70E-14	8.88E-13	8.88E-13	−0.0059
ec00280	Valine, leucine and isoleucine degradation	29	1.86E-11	1.48E-10	4.45E-10	−0.0367
ec00720	Carbon fixation pathways in prokaryotes	26	0.0009	0.0015	0.0216	−0.0369
ec00010	Glycolysis/Gluconeogenesis	34	2.65E-13	3.18E-12	6.37E-12	−0.0416
ec00500	Starch and sucrose metabolism	18	0.0215	0.0257	0.5149	−0.0439
ec00710	Carbon fixation in photosynthetic organisms	15	2.40E-06	1.15E-05	5.77E-05	−0.0607
ec00630	Glyoxylate and dicarboxylate metabolism	18	5.49E-06	2.20E-05	0.0001	−0.0666
ec00260	Glycine, serine and threonine metabolism	30	0.0269	0.0307	0.6456	−0.0724
ec00040	Pentose and glucuronate interconversions	24	0.0160	0.0214	0.3845	−0.0830
ec00020	Citrate cycle (TCA cycle)	23	2.89E-11	1.73E-10	6.93E-10	−0.0867
ec00071	Fatty acid degradation	22	0.0001	0.0003	0.0026	−0.0975
ec00970	Aminoacyl-tRNA biosynthesis	11	0.0022	0.0035	0.0519	−0.1023
ec00072	Synthesis and degradation of ketone bodies	4	0.0208	0.0257	0.4996	−0.1410
ec00983	Drug metabolism - other enzymes	13	0.0425	0.0443	1.0000	−0.1561
ec00230	Purine metabolism	28	0.0002	0.0006	0.0053	−0.1591
ec00250	Alanine, aspartate and glutamate metabolism	16	0.0003	0.0007	0.0080	−0.3449

^1^Pearson correlation of protein and RNA fold changes. Transcriptome data obtained from Fountain *et al*.^30^.

### Correlation analysis between the transcriptome and proteome data

**Figure 5:**
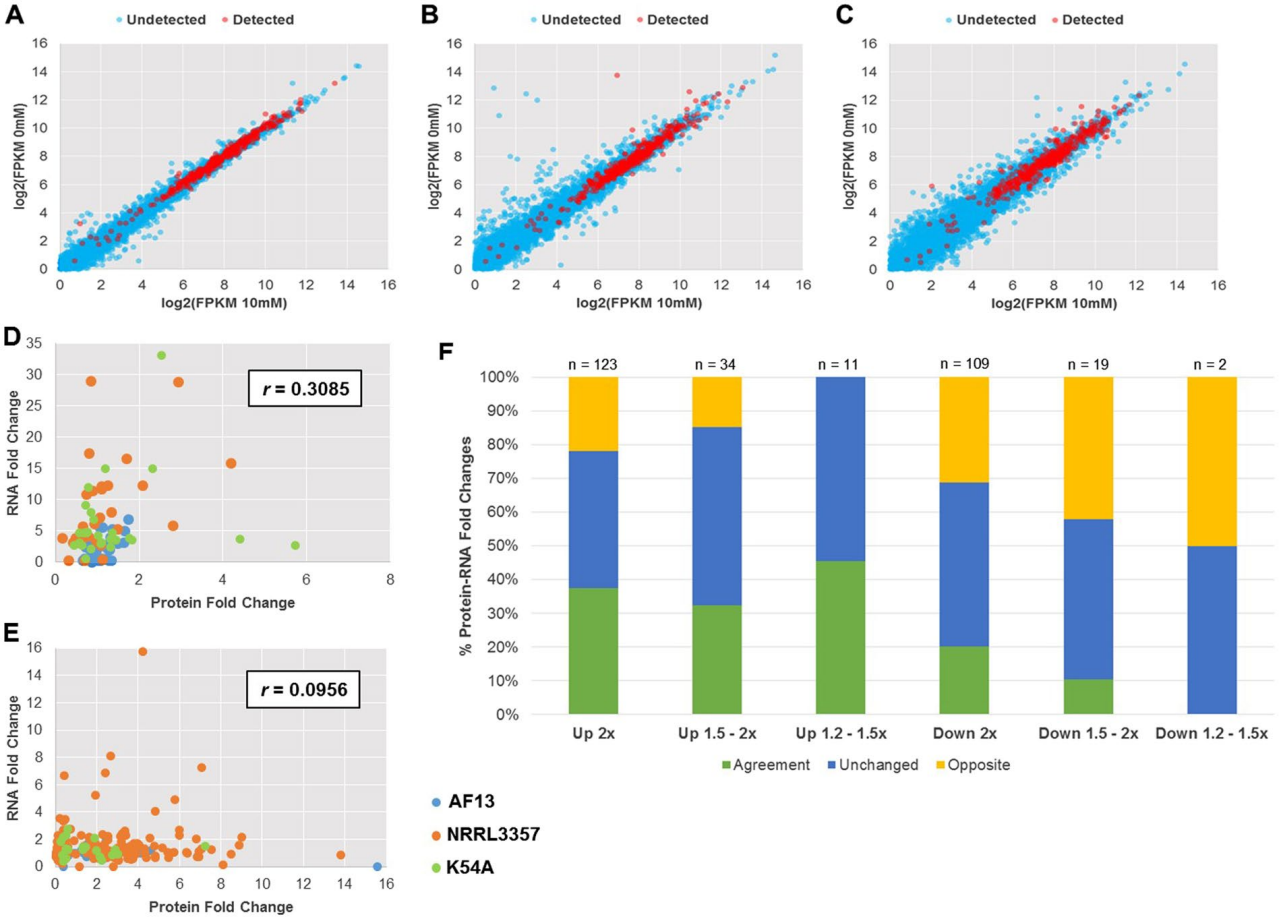
Correlative comparison of iTRAQ proteomics and previously obtained transcriptome data. The expression of transcripts detected (red) and undetected (blue) in the iTRAQ proteomics data were compared between the control and 10 mM H_2_O_2_ treatment for AF13 (**A**), NRRL3357 (**B**), and K54A (**C**) with transcripts exhibiting higher levels of expression being more likely to be detected in the proteomics analysis. Pearson correlations of significantly differentially expressed transcripts with their corresponding proteins (**D**) or significant DEPs with their corresponding transcripts (**E**) showed a low degree of correlation between the datasets. A majority of protein and transcript fold changes showed either agreement or were not changed with regard to up or down regulation and fold regulation with 25.2% showing opposite responses between the datasets (**F**).

Using the available transcriptome data for the examined isolates obtained in our previous study under the same experimental conditions^30,31^, a correlation analysis was performed between expression levels observed in both studies to examine for possible post-transcriptional regulation of oxidative stress responses. Genes exhibiting higher overall levels of expression in each isolate were more likely to be detected in the proteomics analysis (Fig. 5A–C). Pearson correlation of fold changes between all 1,173 detected proteins and their respective transcripts was *r* = 0.0794 with a similar correlation (*r* = 0.0792) observed between the 779 proteins expressed in all biological replicates and their respective transcripts. Pearson correlation between significantly differentially expressed genes (DEGs) and their corresponding proteins were found to be low (*r* = 0.3085) (Fig. 5D). Similarly, correlating the expression observed for significant DEPs to their corresponding transcript expression in the previous transcriptome study resulted in a lower correlation (*r* = 0.0957) (Fig. 5E). Despite a low degree of correlation between the expression levels in the two experiments, the two datasets did show consistency in terms of the trend of changes, with only 25.2% of transcripts showing opposite patterns compared to the DEPs (Fig. 5F).

The degree of correlation varied by pathway and isolate with some pathways showing higher degrees of correlation between the RNA and protein level expression patterns. For example, glutathione metabolism and the pentose phosphate pathway exhibited higher levels of correlation than that observed for the entire dataset (Fig. 5E, Table 2). Conversely, for pathways such as purine metabolism and alanine, aspartate, and glutamate metabolism, negative correlations between the datasets was observed (Table 2). However, examining each pathway correlation by isolate did reveal some variation in the correlation observed. The isolates which expressed greater numbers of DEPs, NRRL3357 and K54A, tended to exhibit higher correlations for each pathway compared to AF13. For example, the glutathione metabolism pathway components in AF13 exhibited a correlation of *r* = 0.1869 while NRRL3357 and K54A exhibited a correlation of *r* = 0.7135 and *r* = 0.4914, respectively (Figure S3).

The pathway correlations observed here (Figure S3), and similarities in overall in protein expression patterns between NRRL3357 and K54A indicated by hierarchical clustering analysis (Figure S2) compared to AF13 may also be indicative of the genetic relationship among these isolates. To examine this, sequencing reads from the previous transcriptome experiment^30,31^ were aligned to the *A. flavus* NRRL3357 reference genome and used for single nucleotide polymorphism (SNP) identification for each isolate. High quality SNPs were used to generate a neighbor joining tree which showed closer genetic relationships among isolates with similar levels of aflatoxin production (Figure S4). NRRL3357 fell within the same clade as AF13, though on a distinct branch from the highly toxigenic isolates AF13 and Tox4. K54A was on a distinct clade from the atoxigenic biological control isolates AF36 and Aflaguard.

### Protein-protein interaction analysis

**Figure 6:**
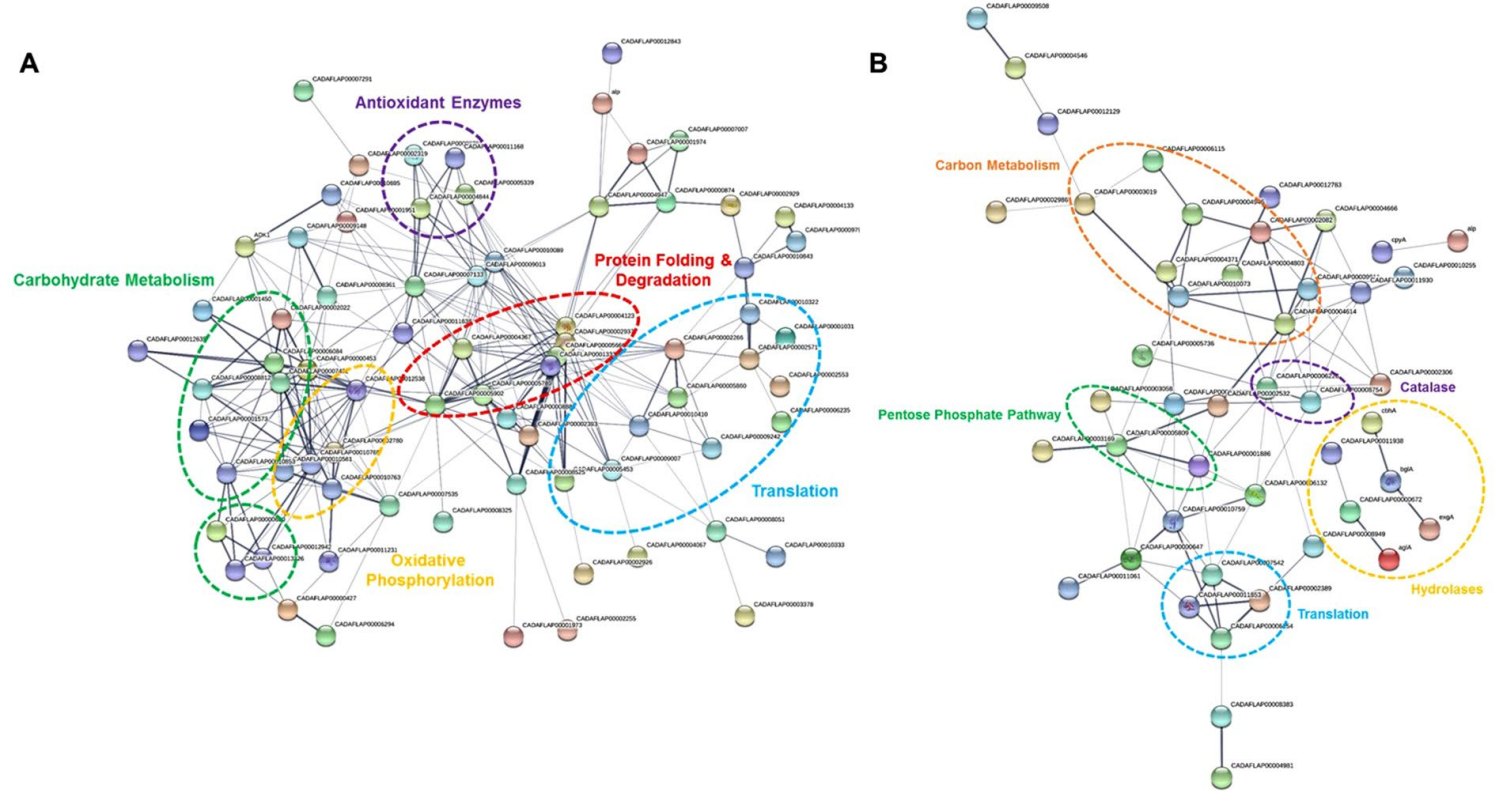
Protein-protein interactions predicted for DEPs found in NRRL3357 in response to increasing oxidative stress. The STRING database was used to examine proteins increased (**A**) or decreased (**B**) in expression in NRRL3357 detected in both H_2_O_2_ treatments. Each node in the network represents a DEP. Interactions are shown by the blue lines connecting each node with the weight of each line representing the confidence of the interaction based on available evidence in the database. Clusters of interest are indicated by the colored labels.

To examine the interactions between DEPs detected in response to oxidative stress in the examined isolates, these proteins were searched for potential physical, co-expression, and co-occurrence types of interactions in the STRING database^38^. For AF13, branches of the interaction network were defined by pathway and molecular function including carbohydrate metabolism and antioxidant enzymes (Figure S5A). For NRRL3357, a more extensive interaction network was obtained than in the other isolates with k-means clustering analysis dividing the network into several key interacting pathway components including antioxidant enzymes, carbohydrate metabolism enzymes, pentose phosphate pathway, oxidative phosphorylation, and translation regulation (Figs 6 and S5B). For K54A, a number of smaller interaction groups were identified and consisted of protein folding, translation, vesicle trafficking, and carbohydrate metabolism pathway components (Figure S5C). Overall, several hub proteins exhibiting physical and co-expression interactions with multiple proteins in diverse pathways were identified in all three isolates. These included protein folding and degradation enzymes such as heat shock proteins (e.g. hsp70 and hsp90), alcohol dehydrogenase (adh1), malate dehydrogenase, G-protein complex proteins (e.g. cpcB), and ATP synthase (α subunit). Additional DEPs not exhibiting interactions with other proteins found in this analysis also likely play indirect roles in oxidative stress responses in isolates of *A. flavus*. A list of all abbreviations and protein names along with the specific node interactions can be found in Table S6.

## Discussion

Aflatoxin production by *A. flavus* and related species of fungi is regulated in concert with other secondary metabolites, developmental processes, and stress-responsive enzymes in response to environmental stress^26,39^. Specifically, oxidative stress has been shown to be a pre-requisite and stimulator of aflatoxin production^22,23^. This is of particular interest given the observation that drought stress results in compromised host resistance to aflatoxin contamination, and that drought stress results in the accumulation of ROS in host plant tissues^16,40,41^. Previously, we have explored differences in oxidative stress responses between field isolates of *A. flavus* at the transcriptome level and found that secondary metabolites, antioxidant enzyme expression, and carbohydrate metabolism may play significant roles in *A. flavus* oxidative stress responses^21,30,31^. In order to further explore these responses at the protein and enzymatic level, here we examined the proteomes of select isolates of *A. flavus* to increasing levels of H_2_O_2_-derived oxidative stress.

Previously, it was found that isolates which produce higher levels of aflatoxin tended to exhibit fewer significant DEGs in response to increasing levels of oxidative stress compared to less toxigenic or atoxigenic isolates^30,31^. At the protein level, the three isolates examined showed similar numbers of DEPs when comparing the control and 10 mM H_2_O_2_ (Table 1). However, comparing at a higher level of stress, 20 or 25 mM H_2_O_2_, the moderately high aflatoxin producing isolate, NRRL3357, exhibited a much greater number of DEPs compared to the high aflatoxin producing isolate, AF13 (Table 1). This suggests a correlation between aflatoxin production levels and oxidative stress tolerance and the vigor of oxidative responses. However, the lack of correlation under moderate stress of DEP numbers and aflatoxin production also implies that other factors in addition to aflatoxin production determine overall stress tolerance. In addition, responses occur at earlier time points not examined in this study are also possible influences.

The lack increasing numbers of DEPs under moderate stress with less stress tolerance and aflatoxin production as seen for DEGs previously^30^ may also be indicative of both experimental variation and post-transcriptional regulation of responses to stress. Post-transcriptional modifications and signaling play a significant role in the regulation of secondary metabolite production and reproductive development in *Aspergillus spp*. and other fungi. For example, phospho-relay signaling networks including MAP kinase components along with G-protein-mediated regulation of aflR through G-protein/cAMP/protein kinase A (pkaA) have been shown to regulate both development and mycotoxin production in *Aspergillus spp*.^31,42–44^. Similar cAMP protein kinase regulators were also found to be differentially expressed here in NRRL3357 under higher levels of oxidative stress (Table S2). Another indication of possible post-transcriptional regulation of protein expression is the low degree of correlation between the transcriptome and proteome data observed here (Fig. 5). Bai *et al*.^34^ observed a similar low degree of correlation (*r* = 0.14) between transcript and protein expression when examining heat stress responses in *A. flavus* which was interpreted as being due to post-transcriptional regulation. Similar levels of correlation between transcriptomic and proteomic data have also been observed in other species including *A. fumigatus* under hypoxic conditions^45^ and *A. nidulans* following long term menadione exposure^46^. While these low degrees of correlation may indeed be due to post-transcription modification and regulation of translation, it is also likely that inherent experimental error due to differences in RNA and protein turnover and biological variation between experimental replicates contribute to such weak correlations^47^.

Comparison of the detected DEPs for each isolate under increasing levels of oxidative stress revealed distinctive, isolate-specific responses with few commonly regulated proteins detected between isolates (Fig. 2B). However, NRRL3357 and K54A did exhibit more commonality than either isolate showed with AF13 despite a more distant genetic relationship between NRRL3357 and K54A compared to NRRL3357 and AF13 (Figs 2B and S4), suggesting a correlation of stress responses with overall oxidative stress tolerance and aflatoxin production capability. The particular DEPs exhibited by the isolates also points to distinct overall strategies with implications for host resistance and interactions with other soilborne microbes. For example, AF13 tended to exhibit increased expression of lytic enzymes such as α-amylase, chitinase, β-glucosidase, glucanase, and α-mannosidase, while NRRL3357 showed decreased expression of the same enzymes, and these were not differentially expressed in K54A (Table S2). These enzymes have been shown to be involved in the colonization of host plant tissues with their inhibitors, such as the α-amylase-inhibiting 14-kDa trypsin inhibitor, being found to accumulate in resistant maize kernel tissues^48–51^. Increased hydrolytic enzyme expression has also been found to provide benefits for fungi in competition with other microbes in soil and plant environments, such as in the case of *Tricoderma spp*. biological controls^52^. These expression levels may also be due to carbon starvation during the transition to a stationary growth phase or with autolysis of hyphal tissues, both of which being likely here given the timepoint examined^53^. However, the production of these enzymes in response to carbon starvation may be beneficial in acquiring new sources of plant-derived carbohydrates in response to starvation and should be studied further^54^.

Differences in the overall strategies employed by the examined isolates could also be seen with regard to primary metabolism. NRRL3357 and K54A tended to show greater degrees of regulation in carbohydrate metabolism and mitochondrial oxidative phosphorylation (Figs 3 and 5, Table S2). Inhibition of oxidative phosphorylation by the application of exogenous compounds such as resveratrol has been shown to compromise fungal oxidative stress tolerance by altering mitochondrial respiration^55^. For carbohydrate metabolism, the active production of glycolysis and TCA cycle intermediates provide the basic components for the biosynthesis of macromolecules, which could be useful in the repair of cellular components with oxidative damage. In addition to glycolysis and the TCA cycle, the pentose phosphate pathway was also stimulated in response to stress in both toxigenic isolates (Fig. 4). This pathway has been shown to be a source of reduced co-factors such as NADPH, which are utilized in the glutathione pathway for non-specific antioxidant activity^56,57^. Interestingly, the moderately toxigenic isolate showed decreased NADPH-generating enzymes in the pentose phosphate pathway which may contribute to reduced stress tolerance compared to the highly toxigenic isolate. Alternatively, this may reflect a reduction in demand for reduced coenzymes due to stress alleviation already provided by other mechanisms at the time point examined.

The differential expression of antioxidant enzyme systems in response to increasing stress may also provide insight into the specific ROS detrimental to these fungi, and to which they are responding. While relatively stable, H_2_O_2_ is also a less reactive ROS resulting in less oxidative damage compared to other, more short lived species such as superoxide (O_2_^−^), hydroperoxyl (HO_2_.), and hydroxyl (OH^−^) radicals^58,59^. Previous studies have explored the effects of specific ROS on aflatoxin production and isolate development in *A. flavus*. For example, Grintzalis *et al*.^24^ found that H_2_O_2_ specifically regulates sclerotia development, and that peroxidized lipids, superoxide, hydroxyl, and thiol radicals tended to have a greater role in aflatoxin production regulation.

Here, we observed that non-specific antioxidant mechanisms such as glutathione metabolism and heat shock proteins tended to be the main ROS scavenging systems increased in response to increasing stress (Table S2, Fig. 6), while catalase was found to be decreased in NRRL3357 under increasing stress (Fig. 6). Given the timepoint examined here, it is possible that the effects of H_2_O_2_-derived stress observed may have been correlated mainly with earlier isolate catalase activity. However, the results do appear to indicate ongoing oxidative stress responses at the studied timepoint. This may also allow for the possibility that other derivative ROS are contributing to the continuing stress the isolates are responding to. Such toxic ROS can be generated non-enzymatically such as through iron cation-mediated interconversions like the Fenton reactions^60^. This coupled with the observations that biosynthetic mechanisms for iron chelating compounds such as kojic acid^30,31,61^ and CPA^62,63^ were regulated in this system (Table S2) suggests that derivative ROS such as OH^−^ may be one of the main causes of oxidative damage in this experiment. In addition, the production of these secondary metabolites may also provide assistance in competition with other soil microbes for iron, a key limiting resource^62^.

In addition to ROS, reactive nitrogen species (RNS) such as nitric oxide (NO) have been found to influence isolate development through carefully timed bursts. Specifically, NO bursts have been found to be involved in the initiation of both conidiation and sclerotia formation in *Aspergillus spp*. and other fungi^64^. This burst of NO is countered by increase in detoxifying enzyme expression, specifically a series of flavohemoproteins^65^. Here, AF13 showed increases in nitric oxide synthase (NOS) and the sclerotia component protein sspA^66^ accompanied by a decrease in flavohemoprotein expression while NRRL3357 showed the opposite pattern (Table S2). This suggests, as was previously hypothesized^34^, that isolate development rates may be influenced by oxidative stress. In addition, the formation of sclerotia is a known survival strategy employed by fungi to cope with inhospitable environmental conditions^24,67,68^. The development of sclerotia indicated by increased sspA expression in AF13 may provide increased environmental stress tolerance compared to the other isolates and may reflect a heightened fitness in competition for soil nutrients.

Isolate secondary metabolism components were also found to be differentially expressed in response to increasing stress. However, very few such proteins were detected in this experiment likely due to either limitations of the protein isolation or iTRAQ protocols employed^69^, or due to time dependent regulation of expression given that the majority of aflatoxin production occurs 2–6 days in stationary culture^70^. Previously, we observed that the moderately toxigenic isolate, NRRL3357 showed significant increases in aflatoxin gene expression under high levels of oxidative stress^30,31^ which corresponded to the observed increase in ver-1 expression here (Table S1). It has been proposed that the production of secondary metabolites such as aflatoxin may provide supplemental antioxidant protection to *A. flavus* and other Aspergilli. While there is no evidence that aflatoxin itself functions as an antioxidant, the biochemical process of producing aflatoxins has been shown to contribute to oxidative stress tolerance with deletion of pathway components causing reduced conidial oxidative stress tolerance^71^. This antioxidant benefit hypothesized to occur during aflatoxin production either through the consumption of excess molecular oxygen, or through the stimulation of antioxidant enzyme expression through localized secondary ROS bursts^23,26,30,71^. Overall, the aflatoxin production capabilities of the examined isolates correlates with each individual isolate’s expression of competitive and pathogenicity-related proteins, and their ability to grow under higher levels of oxidative stress (Table S2)^21,30,31^. This suggests that aflatoxin production may contribute to isolate competitiveness and/or oxidative stress tolerance. The overall trends observed in the data are summarized in Figure S6.

The regulation of aflatoxin biosynthetic components has serious implications for host resistance. Under drought stress, it has been shown that drought sensitive, aflatoxin contamination susceptible varieties of maize accumulate higher levels of ROS and RNS under drought compared to drought tolerant, aflatoxin resistant varieties^40,41^. Given this correlation, the observed stimulation of aflatoxin production by ROS^21–23^, and the role of RNS and nitrogen availability in fungal development and mycotoxin production regulation^64,65,72–74^, the responses of *A. flavus* to drought-derived oxidative stress may provide insights for enhancing host resistance.

Current molecular breeding practices have been successful in developing both maize and peanut lines with degrees of aflatoxin resistance and drought tolerance^15^. While progress has been made, additional measures will be necessary to further enhance available resistance to aflatoxin contamination. Utilizing novel advances in biotechnology such as transgenic and genome editing approaches, the expression of lytic enzyme inhibiters and antioxidant enzymes may be enhanced to counter pathogenicity factors produced by highly competitive and toxigenic fungi, and to alleviate drought-related ROS accumulation to reduce cellular damage and the stimulation of additional aflatoxin production. Other approaches such as RNA interference and host-induced gene silencing (HIGS) of aflatoxin biosynthetic genes are also possible^75^. These approaches provide a future direction for enhancing both drought tolerance and aflatoxin contamination resistance in both maize and peanut.

## Materials and Methods

### Isolate collection

The AF13 isolate used in this study was obtained from Dr. Kenneth Damann, Department of Plant Pathology and Crop Physiology, Louisiana State University, Baton Rouge, LA. The NRRL3357 isolate was obtained from the USDA National Culture Repository, Peoria, IL. The K54A isolate was obtained from Dr. Hamed Abbas, USDA-ARS, Mycotoxin Res Unit, Stoneville, MS. All isolates were received on potato dextrose agar (PDA) and were sub-cultured on V8 agar (20% V8 juice, 1% CaCO_3_, 2% agar) prior to use.

### Isolate culture conditions

Conidia of each isolate were harvested from V8 agar plates five days after inoculation using sterile 0.1% Tween 20 solution. The isolates were then cultured in 125 mL Erlenmeyer flasks containing 50 mL yeast extract-sucrose (YES; 2% yeast extract, 1% sucrose) medium inoculated with 100 µL conidia suspension (~4.0 × 10^6^ conidia/mL). For oxidative stress treatments, the YES medium was amended with H_2_O_2_ (3% stabilized solution) at concentrations as previously determined based on individual isolates’ oxidative stress tolerance^21^ with AF13 cultures amended with 0, 10, and 25 mM H_2_O_2_; NRRL3357 with 0, 10, and 20 mM H_2_O_2_; and K54A with 0 and 10 mM H_2_O_2_. The isolates were then stationary cultured at 30 °C for 7 days in the dark. Three biological replicate cultures were performed for each isolate and treatment combination. Following culturing, mycelia was recovered and stored at −80 °C for protein isolation.

### Protein isolation and quantitation

Proteins were isolated using a modified phenol/methanolic ammonium acetate method based on Zhuang *et al*.^76^ and Hurkman and Tanaka^77^. Briefly, the obtained mycelia tissue was ground to a fine powder using a mortar and pestle cooled in liquid nitrogen. The powdered tissue (200 mg) was then incubated in extraction media (0.1 M Tris-HCL pH 8.8, 10 mM EDTA, 1.2% β-mercaptoethanol (*v*/*v*), 0.9 M Sucrose) for 20 min on ice with occasional vortexing. Tris-buffered phenol (pH 8.8) was then added and the samples incubated in ice for a further 10 min. Following centrifugation at 5,000× g at 4 °C for 20 min, proteins in the phenol phase were then precipitated in a separate tube in five volumes of cold 0.1 M ammonium acetate in 100% methanol. The precipitated proteins were then pelleted by centrifugation and washed twice in both 0.1 M ammonium acetate and then in cold 80% acetone. The pellet was then dissolved in 2D buffer (8 M Urea, 4% CHAPS (*w*/*v*), 40 mM Tris-base, 2 M Thiourea) immediately prior to quantitation using an EZQ Protein Quantitation Kit (Invitrogen, Carlsbad, CA, USA) according to the manufacturer’s instructions. One-dimensional polyacrylamide gel electrophoresis was then performed to validate protein quality.

### Protein digestion and iTRAQ labeling

Following quantitation, for each sample 100 µg of protein was dissolved in the dissolution buffer containing denaturant found in the iTRAQ Reagents –8-plex kit (AB Sciex Inc., Foster City, CA, USA) then reduced, alkylated, digested with trypsin, and labeled according to the manufacturer’s instructions. For the AF13 isolate, the 0, 10, and 25 mM H_2_O_2_ treated samples were labeled with iTRAQ tags 113, 114, and 115, respectively. For the NRRL3357 isolate, the 0, 10, and 20 mM H_2_O_2_ treated samples were labeled with tags 116, 117, and 118, respectively. Finally, for the K54A isolate, the 0, and 10 mM H_2_O_2_ treated samples were labeled with tags 119 and 121, respectively. Each of the three biological replicates were processed separately with the same labeling strategy. Following labeling, each biological replicate of samples were mixed and aliquoted into four technical replicates.

### Strong cation exchange fractionation, reverse phase nanoflow HPLC, and tandem mass spectrometry

Peptide fractionation, HPLC, and mass spectrometry were performed as described in our previous study^78^. Briefly, each peptide mixture was lyophilized and dissolved in Solvent A (25% acetonitrile (*v*/*v*), 10 mM ammonium formate, pH 2.8) followed by fractionation on a Agilent HPLC System 1260 (Agilent Technologies, Santa Clara, CA, USA) with a polysulfoethyl column (2.1 mm × 100 mm, 5 µL, 300 Å; PolyLC, Columbia, MD, USA). Elution was performed with a flow rate of 200 µL/min with a linear gradient of 0–20% Solvent B (25% acetonitrile, 0.5 M ammonium formate, pH 6.8) over 50 min. Ramping up was then performed with 100% Solvent B for 5 min. Absorbance at 280 nm was monitored, and a total of 10 fractions were collected. The collected fractions were then resuspended in LC solvent A (0.1% formic acid in 3% acetonitrile) and used for analysis on a hybrid quadrupole Orbitrap (Q Exactive Plus) MS system (Thermo Fisher, Bremen, Germany) coupled with an Easy-nLC 1000 system (Thermo Fisher). Mass analysis was performed in positive ion mode with high collision dissociation energy. The scan range was 400–2,000 m/z with full MS resolution of 70,000 and 200–2,000 m/z with MS2 resolution of 17,500. The first mass was fixed at 115 m/z, and 445.12003 m/z (polysiloxane ion mass) was used for real-time mass calibration. Mass spectral data obtained in this study have been deposited in the ProteomeXchange repository with the dataset identifier PXD007164^79^.

### Peptide identification, relative quantification, and bioinformatics analysis

Peptide sequences were identified based on the obtained MS/MS data using the ProteinPilot (v4.5) software (Applied Biosystems) against specified non-redundant databases (combined Uniprot, http://www.uniprot.org/uniprot/?query=aspergillus+flavus&sort=score; NCBI, https://www.ncbi.nlm.nih.gov/gquery/?term=aspergillus+flavus). Data normalization was performed using default settings along with differential expression and p-value estimation using ProteinPilot. An expression change was considered as significant only when the protein fold change was quantified as ≥1.2 or ≤0.8 with *p* ≤ 0.05 in at least two of three biological replicates, along with a Fisher’s combined probability (*q*-value) of ≤0.05^80^.

Functional classification and subcellular localization of the detected and differentially expressed proteins was performed using the Gene Ontology (GO) enrichment tool in FungiDB^35^. Functional and localization term consolidation was then performed using REVIGO^36^. Visualization of GO biological process terms for each isolate was performed based on REVIGO outputs using R-studio and R (v3.3.0). Venn diagrams of differentially expressed proteins were created using Venny (v2.1). Heatmaps of expression patterns, hierarchical clustering analysis, and principal components analysis (PCA) were performed using the Multi-experiment Viewer (MeV; v4.9.0)^81^. Biological pathway enrichment analysis was performed using the metabolic pathway analysis tool in FungiDB using the Kyoto Encyclopedia of Genes and Genomes (KEGG)^37^. For the genetic analysis, BAM files for each isolate used in the previous transcriptome study^30,31^ under the same experimental conditions used here were merged using Samtools (v1.6)^82,83^. BAM improvement was performed according to recommended practices using Picard-tools (http://broadinstitute.github.io/picard). Following improvement, variant calling was performed using Bcftools (v1.6) mpileup^82,83^. Raw variants were then filtered to remove low quality SNPs and missing data using Bcftools (v1.6; Quality ≥10, Depth ≥10, MQ ≥30, and Missing Data ≤2). TASSEL (v5)^84^ was then used to construct the neighbor joining tree based on the observed variants. Finally, predicted protein-protein interactions were examined using STRING (v10.5)^38^.

### Data availability

MS data have been deposited in the ProteomeXchange repository with the dataset identifier PXD007164.

## Electronic supplementary material

Supplementary Information
