# Supplementary material for: Proteome analysis of Aspergillus flavus isolate-specific responses to oxidative stress in relationship to aflatoxin production capability
Source: Sci Rep. 2018 Feb 21;8:3430. doi: 10.1038/s41598-018-21653-x (PMC5821837; doi:10.1038/s41598-018-21653-x)
Supplement: Supplementary file 1 — Supplementary Information [file 41598_2018_21653_MOESM1_ESM.pdf]

Proteome analysis of *Aspergillus flavus* isolate-specific responses to oxidative stress in relationship to aflatoxin production capability

Supplemental Information

Jake C. Fountain[1,2], Jin Koh[3], Liming Yang[1,2,4], Manish K. Pandey[5], Spurthi N. Nayak[5], Prasad Bajaj[5], Wei-Jian Zhuang[6], Zhi-Yuan Chen[7], Robert C. Kemerait[1], R. Dewey Lee[8], Sixue Chen[3], Rajeev K. Varshney[5], Baozhu Guo[2,*]

[1] Department of Plant Pathology, University of Georgia, Tifton, GA, USA

[2] USDA-ARS Crop Protection and Management Research Unit, Tifton, GA, USA

[3] Interdisciplinary Center for Biotechnology Research, University of Florida, Gainesville, FL, USA

[4] School of Life Sciences, Huaiyin Normal University, Huaian, Jiangsu, China

[5] International Crop Research Institute for the Semi-Arid Tropics (ICRISAT), Patancheru, Telangana, India

[6] College of Life Sciences, Fujian Agriculture and Forestry University, Fuzhou, Fujian, China

[7] College of Plant Protection, Fujian Agriculture and Forestry University, Fuzhou, Fujian, China

***Corresponding Author:**
Dr. Baozhu Guo
baozhu.guo@ars.usda.gov

Supplemental Figures

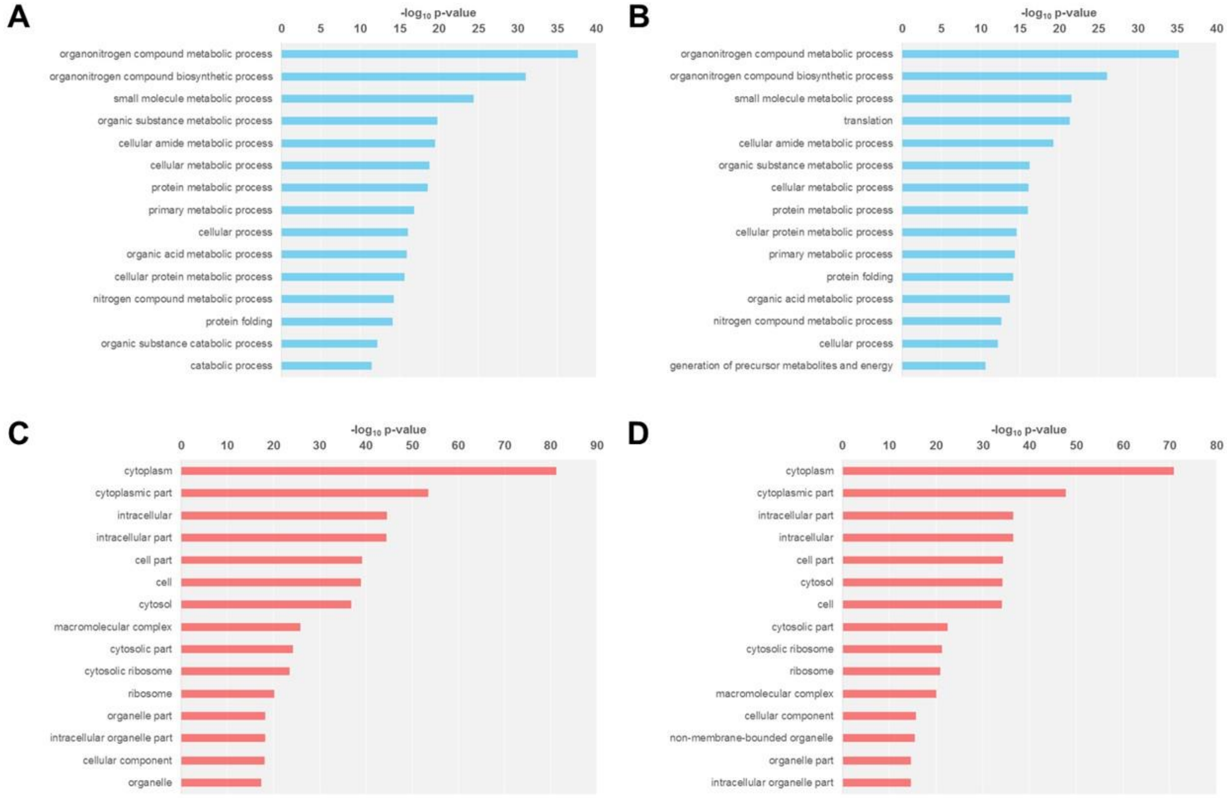

Figure S1. Gene ontology (GO) enrichment analysis by replication. Gene ontology (GO) analysis for biological function (A, B) and cellular localization (C, D) was performed for all 1,173 proteins detected (A, C), and for the 799 proteins detected in all three biological replicates of the study (B, D). The plots show the top 15 GO categories sorted in decreasing order by $-\log_{10}$ (p-value). Overall, the results between the two data sets were very similar with most of the top enriched GO terms held in common for both biological function and cellular localization.

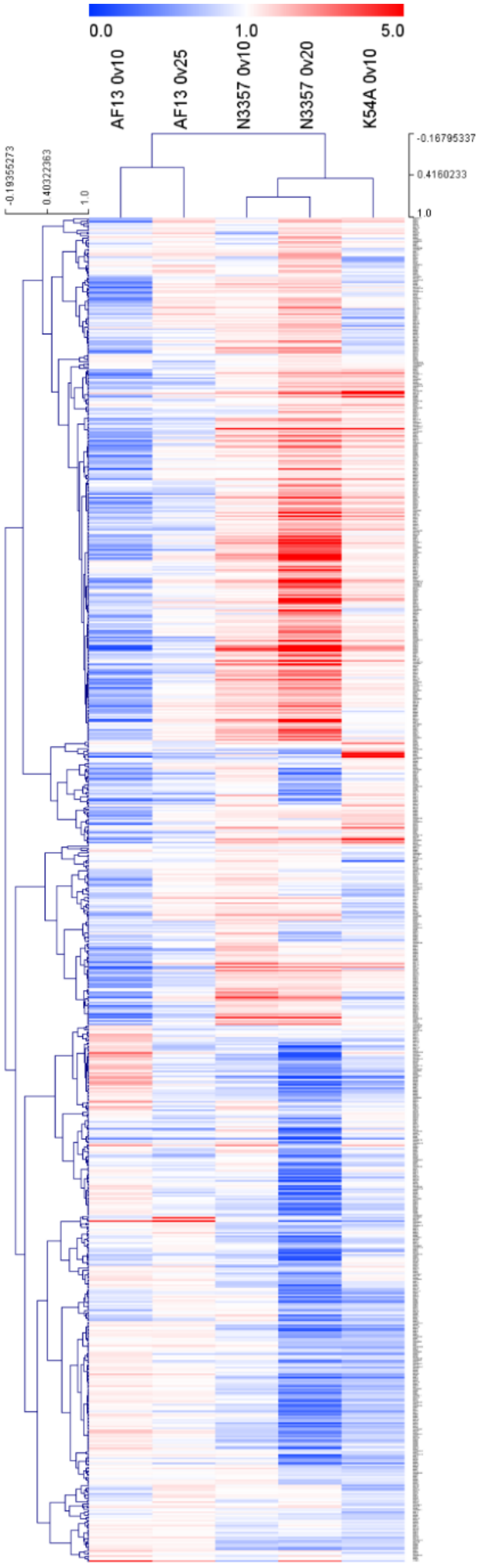

0.0
1.0
5.0
AF13 0v10
AF13 0v25
N3357 0v10
N3357 0v20
K54A 0v10
-0.16795337
0.4160233
1.0
-0.19355273
0.40322363
1.0

Figure S2. Heatmap and hierarchical clustering analyses of protein relative expression patterns. The heatmap represents the observed fold changes in expression for the 799 proteins found to be expressed in all three biological replicates of the experiment. Blue color represents decreased patterns. Red color represents increased patterns. Hierarchical clustering analysis of the proteins shows several distinct clades with unique expression patterns showing isolate-specific responses to stress. Overall differences in isolate expression can also be visualized in the sample-level tree.

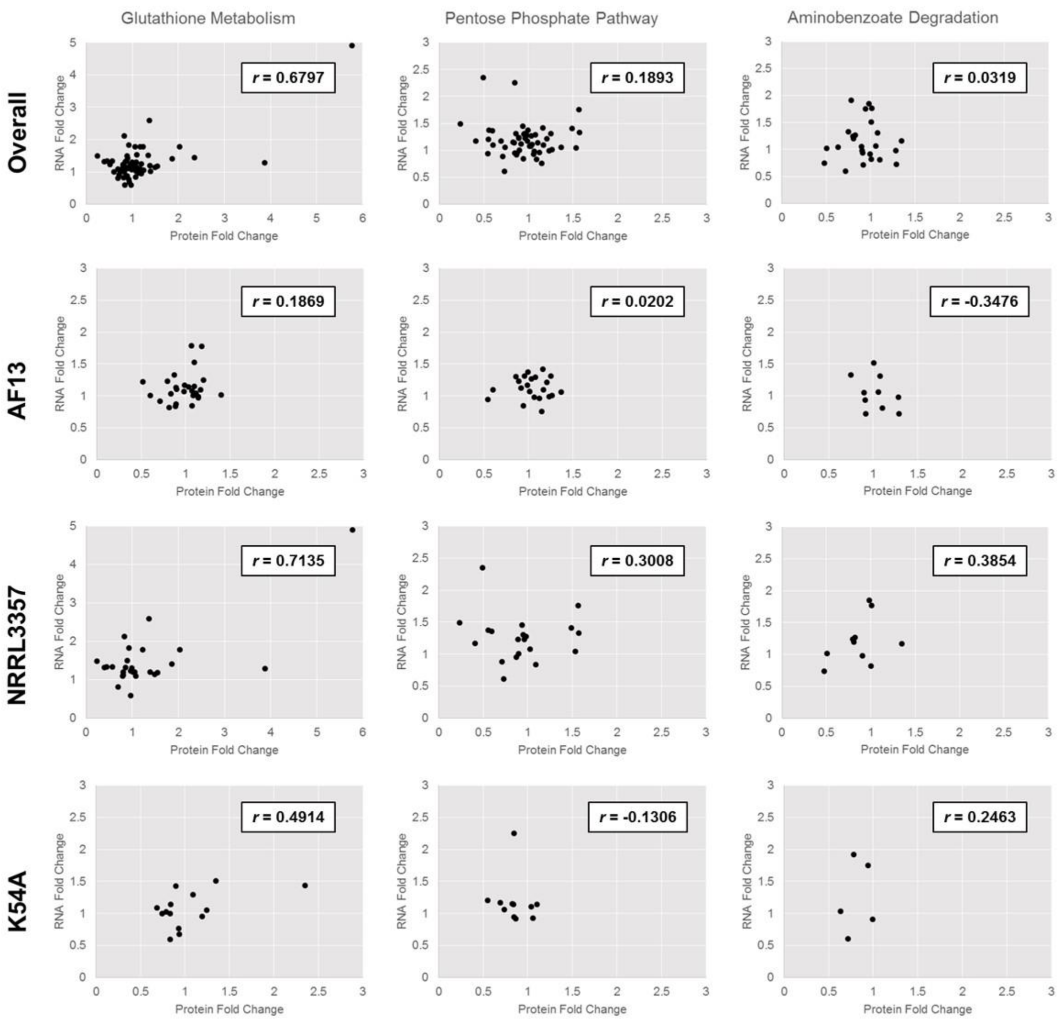

Figure S3. Pearson correlations of proteome and transcriptome data for select metabolic pathways. Pearson correlations of detected protein expression with corresponding transcripts when comparing the control and 10mM H_2O_2 treatments were performed for proteins involved in glutathione metabolism, the pentose phosphate pathway, and aminobenzoate degradation for all isolates (overall plots) and for each individual isolate. Greater degrees of correlation could be observed within each pathway both overall and within each isolate than observed for all proteins and transcripts. Isolate-to isolate variation in correlation was also observed within each pathway likely corresponding to the degree of differential expression observed for the proteins and transcripts in each pathway.

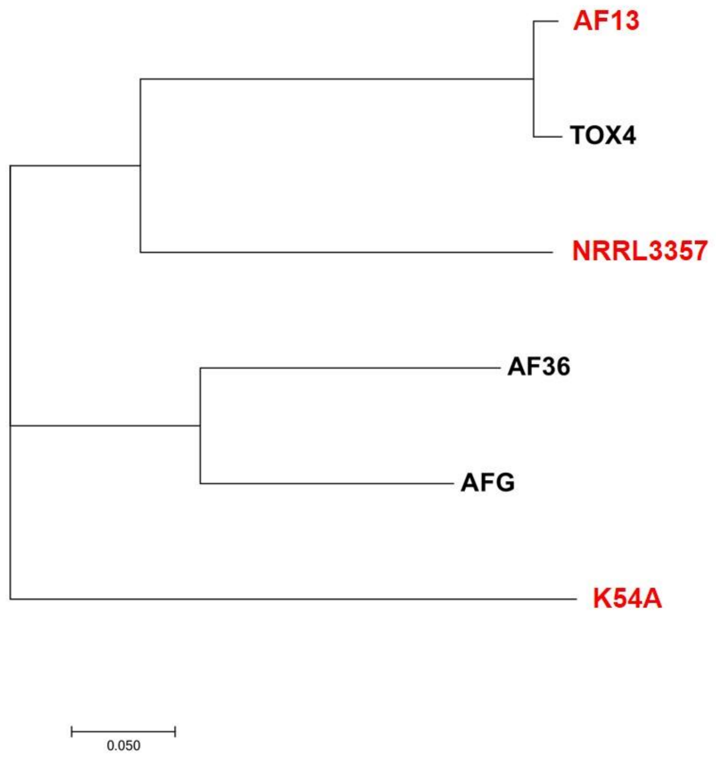

Figure S4. Neighbor joining tree analysis of isolates examined using proteomic and/or transcriptomic analyses. Transcriptome sequencing reads obtained in the previous experiment [30,31] were used to call high-quality SNPs for each isolate under the same experimental conditions used in the present study. These SNPs were used to build a neighbor joining tree to visualize the genetic relationship between these isolates. Isolates used in the present study are highlighted in red.

A

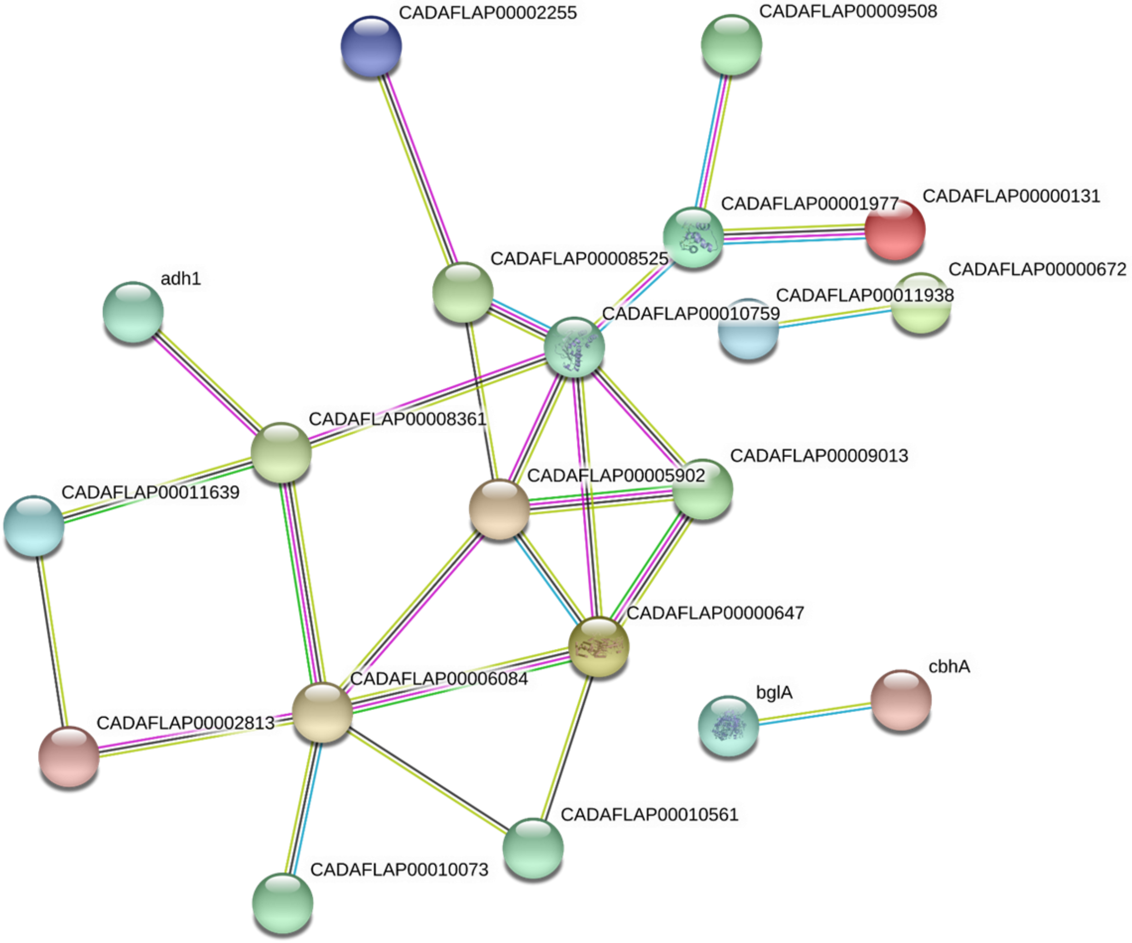
CADAFLAP00002255
CADAFLAP00009508
CADAFLAP00001977
CADAFLAP00000131
CADAFLAP00008525
CADAFLAP00000672
adh1
CADAFLAP00011938
CADAFLAP00010759
CADAFLAP00008361
CADAFLAP00009013
CADAFLAP00005902
CADAFLAP00011639
CADAFLAP00000647
cbhA
CADAFLAP00006084
bglA
CADAFLAP00002813
CADAFLAP00010561
CADAFLAP00010073

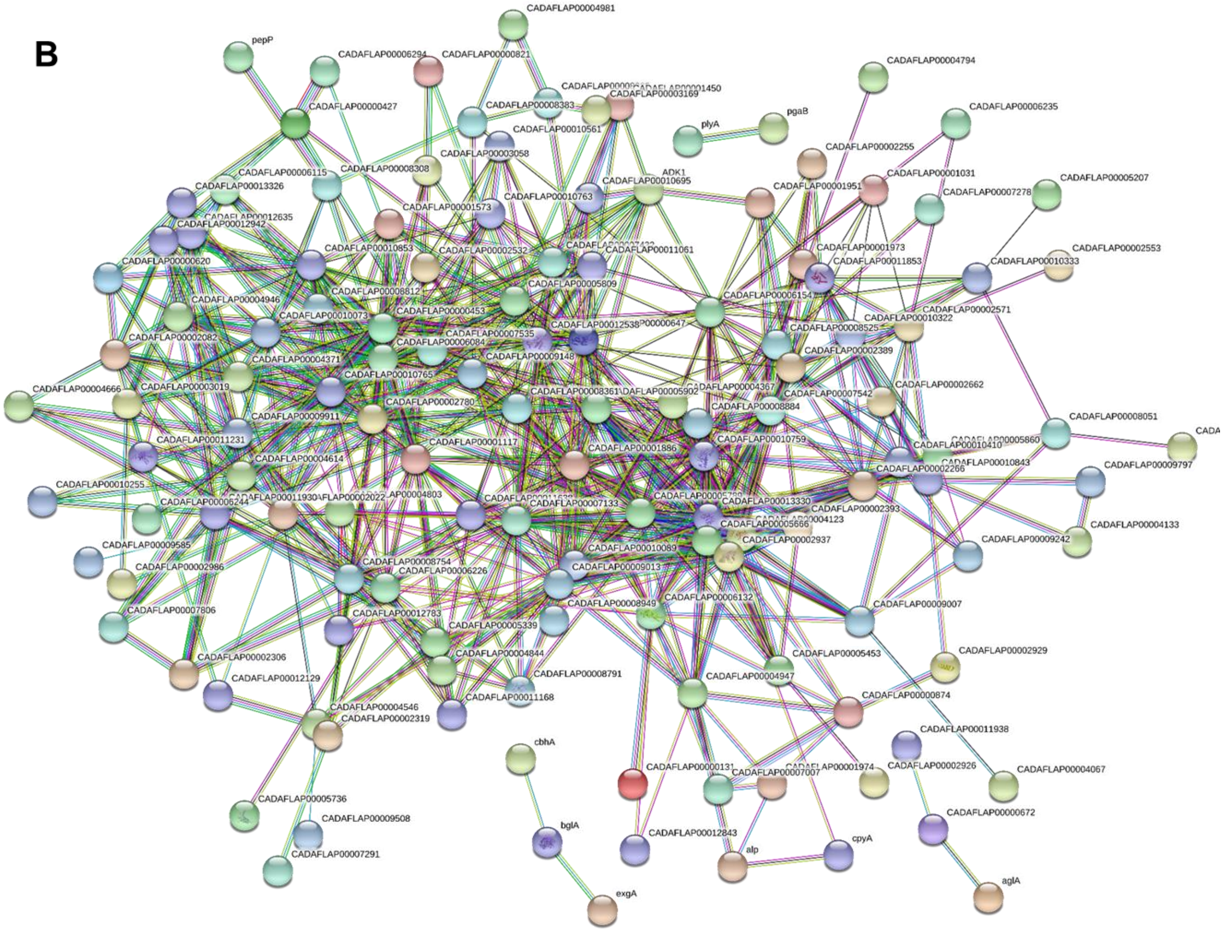
B
CADAFLAP00004981
pepP
CADAFLAP00006294
CADAFLAP00000821
CADAFLAP00004794
CADAFLAP00003169
CADAFLAP00000427
CADAFLAP00008383
CADAFLAP00010561
plyA
pgaB
CADAFLAP00006235
CADAFLAP00003058
CADAFLAP00002255
CADAFLAP00006115
CADAFLAP00008308
ADK1
CADAFLAP00010695
CADAFLAP00001031
CADAFLAP00005207
CADAFLAP00013326
CADAFLAP00010763
CADAFLAP00001951
CADAFLAP00007278
CADAFLAP00012635
CADAFLAP00001573
CADAFLAP00012942
CADAFLAP00010853
CADAFLAP00002532
CADAFLAP00011061
CADAFLAP00001973
CADAFLAP00002553
CADAFLAP00000620
CADAFLAP00011853
CADAFLAP00010333
CADAFLAP00008812
CADAFLAP00005809
CADAFLAP00004946
CADAFLAP00006154
CADAFLAP00002571
CADAFLAP00010073
CADAFLAP00000453
CADAFLAP00012538
CADAFLAP00010322
CADAFLAP00007535
CADAFLAP00008525
CADAFLAP00002082
CADAFLAP00006084
CADAFLAP00002389
CADAFLAP00004371
CADAFLAP00009148
CADAFLAP00004367
CADAFLAP00010765
CADAFLAP00002662
CADAFLAP00004666
CADAFLAP00003019
CADAFLAP00005902
CADAFLAP00007542
CADAFLAP00008884
CADAFLAP00009911
CADAFLAP00002780
CADAFLAP00008051
CADAFLAP00010759
CADAFLAP00005860
CADAFLAP00011231
CADAFLAP00001117
CADAFLAP00001886
CADAFLAP00010410
CADAFLAP00004614
CADAFLAP00010843
CADAFLAP00009797
CADAFLAP00002266
CADAFLAP00010255
CADAFLAP00004803
CADAFLAP00006244
CADAFLAP00007133
CADAFLAP00013330
CADAFLAP00002393
CADAFLAP00004123
CADAFLAP00004133
CADAFLAP00005666
CADAFLAP00009585
CADAFLAP00002937
CADAFLAP00009242
CADAFLAP00010089
CADAFLAP00002986
CADAFLAP00008754
CADAFLAP00009013
CADAFLAP00006226
CADAFLAP00007806
CADAFLAP00008949
CADAFLAP00006132
CADAFLAP00009007
CADAFLAP00012783
CADAFLAP00005339
CADAFLAP00004844
CADAFLAP00005453
CADAFLAP00002929
CADAFLAP00002306
CADAFLAP00008791
CADAFLAP00012129
CADAFLAP00004947
CADAFLAP00011168
CADAFLAP00000874
CADAFLAP00004546
CADAFLAP00002319
CADAFLAP00011938
cbhA
CADAFLAP00000131
CADAFLAP00007007
CADAFLAP00001974
CADAFLAP00002926
CADAFLAP00004067
CADAFLAP00005736
CADAFLAP00009508
CADAFLAP00000672
bglA
CADAFLAP00012843
cpyA
alp
CADAFLAP00007291
aglA
exgA

C

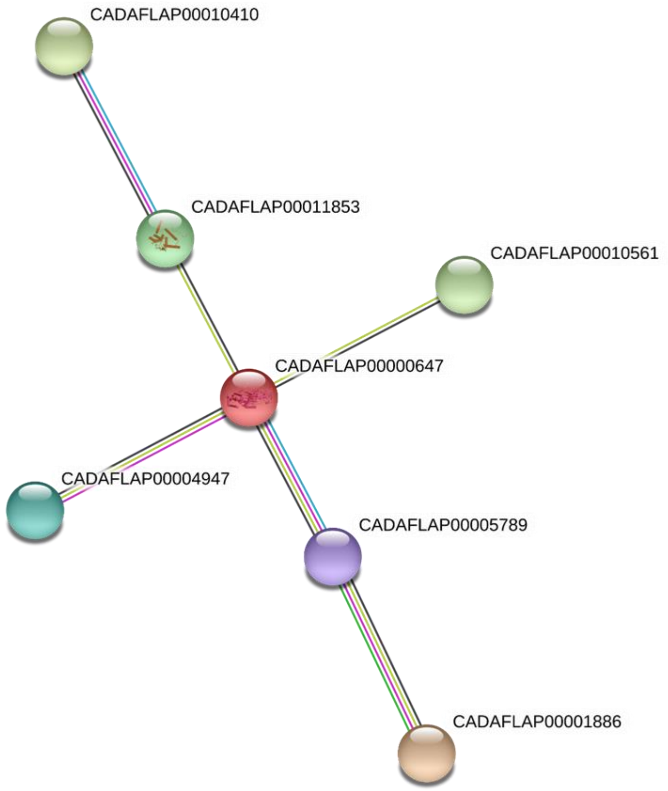

Figure S5. STRING interaction networks for differentially expressed proteins observed in each isolate in response to increasing levels of oxidative stress. STRING interaction plots for AF13 (A), NRRL3357 (B), and K54A (C) were performed. Nodes are colored based on software default parameters. Distance between nodes and their position relative to each other indicate their relatedness in terms of biological function, protein-protein interaction, and co-expression. Lines connecting nodes represent different forms of evidence for a relationship between the proteins connected by the nodes. Of note are the following colored lines representing available evidence of interactions: light blue: known interactions from curated databases; pink: experimentally determined interactions; dark blue: gene co-occurrence; yellow: textmining; and black: co-expression.

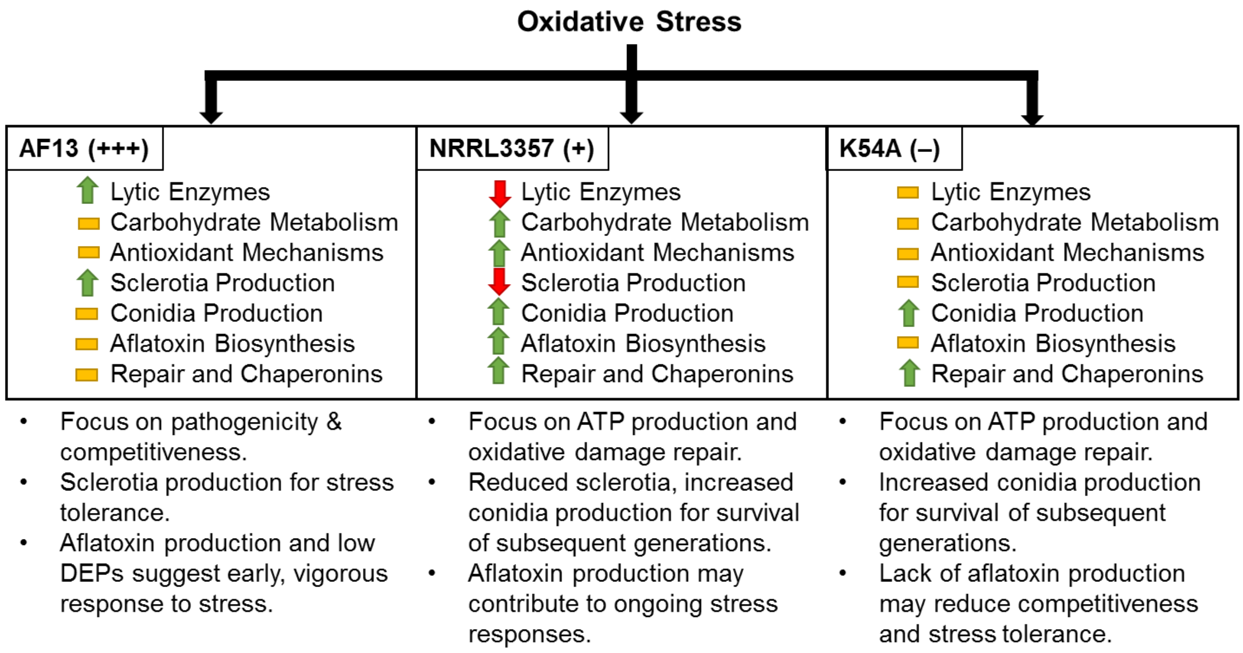

Figure S6. Summary of isolate oxidative stress responses at the protein level. Summary of DEPs observed in key biological pathways in each isolate under increasing oxidative stress. The green and red arrows correspond to increased and decreased expression, respectively, relative to the control. The yellow bars represent no significant changes in expression under increasing stress. Overall, AF13 exhibited increased expression of lytic enzymes and sclerotial production proteins which likely contributes to increased pathogenicity, competitiveness, and ability to endure environmental stress. Elevated aflatoxin production and stress tolerance is also likely the produce of earlier, vigorous responses not observed in the present study. Both NRRL3357 and K54A focused on oxidative damage repair and ATP generation to provide for ongoing stress responsive processes. Increased conidia production suggested by protein expression was also observed in our previous study [21] and is likely the result of efforts to ensure survival of the next generation. The lack of aflatoxin production may contribute to less stress tolerance and competitiveness in K54A.

Table S1. Summary of generated iTRAQ proteomics data

Data	*FDR Type*	FDR	Rep 1	Rep 2	Rep 3	Average
Proteins	Global	1%	2,147	1,907	1,646	1,900
Distinct Peptides	Global	1%	19,936	17,479	17,677	18,364
Spectra	Global	1%	67,246	66,352	74,767	69,455

Table S2. Differentially expressed proteins in *A. flavus* isolates in response to oxidative stress.

Gene ID	Uniprot ID	Annotation	Fold Change	*q* -value
		**AF13 0 v 10mM**		
AFLA_095660	B8NKY3	Immunoglobulin G-binding protein H, putative	4.1150	<0.0001
AFLA_021870	A0A0D9MMH9	Glucanase	3.6492	<0.0001
AFLA_026140	B8N0S9	Alpha-amylase	2.9625	0.0001
AFLA_033400	A0A0D9N9J1	alpha-1,2-Mannosidase	2.4351	0.0014
AFLA_045980	A0A0D9N070	Prolyl oligopeptidase family protein	2.3179	<0.0001
AFLA_006960	B8NQL4	Molecular chaperone and allergen Mod-E/Hsp90/Hsp1	1.8458	<0.0001
AFLA_125270	A0A0D9MSQ9	Uncharacterized protein	1.8241	<0.0001
AFLA_078900	B8MXV3	Beta-hexosaminidase	1.7422	<0.0001
AFLA_124500	B8NML4	Nitric oxide synthase, putative	1.6632	<0.0001
AFLA_027070	B8N394	Acetyl-coenzyme A synthetase	1.5870	0.0005
AFLA_130150	A0A0D9NA25	Uncharacterized protein	1.5467	<0.0001
AFLA_102010	B8NU30	Class V chitinase, putative	1.5449	<0.0001
AFLA_057770	B8NE46	Probable pectate lyase A	1.5206	0.0007
AFLA_097750	C5H8J2	SspA	1.5000	0.0044
AFLA_051140	A0A0D9MWN4	Beta-glucosidase	1.4575	0.0002
AFLA_034380	B8N244	Catalase, putative	0.6157	<0.0001
AFLA_122720	A0A0D9MR79	Variant SH3 domain protein	0.5557	0.0039
AFLA_071010	B8NK52	Heat shock protein (Sti1), putative	0.5549	0.0001
AFLA_026020	Q52QR9	Calmodulin A	0.4990	0.0043
AFLA_022480	B8N5C0	26S proteasome regulatory particle subunit Rpn8, putative	0.4902	0.0003
AFLA_099650	B8NTN4	Woronin body major protein, putative	0.4431	<0.0001
AFLA_063320	B8NFJ8	Uncharacterized protein	0.4424	<0.0001
AFLA_045750	B8NBK0	Antigenic mitochondrial protein HSP60, putative	0.4044	0.0011
AFLA_070490	B8NK00	Uncharacterized protein	0.3927	0.0007
AFLA_093280	B8NKS4	Disulfide isomerase (TigA), putative	0.3892	0.0005
AFLA_056260	B8ND65	Nascent polypeptide-associated complex (NAC) subunit, putative	0.3336	0.0007
AFLA_042700	A0A0D9MTF9	RNA recognition motif	0.2996	0.0012
AFLA_133940	B8NFW5	Uncharacterized protein	0.2663	<0.0001
AFLA_054750	B8ND04	Malate dehydrogenase, NAD-dependent	0.2599	0.0001
AFLA_060110	B8NEA2	Uncharacterized protein	0.2151	<0.0001
AFLA_073480	B8MW01	Tropomyosin, putative	0.1945	0.0005
AFLA_050270	B8NSS6	Conserved lysine-rich protein, putative	0.1869	<0.0001
AFLA_088570	B8MWG7	Uncharacterized protein	0.1776	0.0002
AFLA_031780	B8N3U4	Telomere and ribosome associated protein Stm1, putative	0.1715	<0.0001
AFLA_117640	B8NVS7	CipC-like antibiotic stress responsive protein	0.1645	<0.0001
AFLA_093220	B8NKR8	Ran-specific GTPase-activating protein 1, putative	0.1473	0.0014
AFLA_007700	B8NXF1	Uncharacterized protein	0.1456	<0.0001
AFLA_069370	B8NIQ9	Phosphoglycerate kinase	0.1434	<0.0001
AFLA_052860	B8NS55	Chaperone/heat shock protein Hsp12, putative	0.1170	<0.0001
AFLA_028800	B8N3H7	Eukaryotic translation initiation factor 5A	0.0783	0.0001
		**AF13 0 v 25mM**		
AFLA_095660	B8NKY3	Immunoglobulin G-binding protein H, putative	15.5847	<0.0001
AFLA_048690	A0A0D9N9P4	Alcohol dehydrogenase GroES-like domain protein	4.5965	0.0013
AFLA_004950	B8NQA4	Cytochrome c oxidase subunit Va, putative	2.1802	0.0009

Table S2. Cont.

Gene ID	Uniprot ID	Annotation	Fold Change	*q* -value
AFLA_121090	B8NWS2	Uncharacterized protein	2.0621	0.0051
AFLA_099010	B8NUD9	Transaldolase	1.7166	<0.0001
AFLA_078650	B8MXS8	ATP synthase subunit alpha	1.6139	0.0010
AFLA_039410	B8NCN4	Cell wall serine-threonine-rich galactomannoprotein Mp1	1.3686	0.0001
AFLA_119460	A0A0D9MQY3	NAD dependent epimerasedehydratase family protein	1.3553	0.0026
AFLA_071350	B8NJ18	UDP-N-acetylglucosamine pyrophosphorylase	1.3545	0.0010
AFLA_006960	B8NQL4	Molecular chaperone and allergen Mod-E/Hsp90/Hsp1	1.2394	0.0003
AFLA_076170	B8MX30	Uncharacterized protein	0.9241	0.0001
AFLA_034380	B8N244	Catalase, putative	0.5908	<0.0001
AFLA_007700	B8NXF1	Uncharacterized protein	0.5875	<0.0001
AFLA_060110	B8NEA2	Uncharacterized protein	0.4245	<0.0001
		**NRRL3357 0 v 10mM**		
AFLA_112130	A0A0D9MS17	Hydrophobic surface binding protein A	6.9512	0.0001
AFLA_054750	B8ND04	Malate dehydrogenase, NAD-dependent	4.3005	<0.0001
AFLA_007700	B8NXF1	Uncharacterized protein	3.6045	<0.0001
AFLA_097190	B8NU47	Uncharacterized protein	3.1380	0.0003
AFLA_069370	B8NIQ9	Phosphoglycerate kinase	3.0778	0.0003
AFLA_130310	B8NPF9	Protein disulfide-isomerase	2.9678	<0.0001
AFLA_045750	B8NBK0	Antigenic mitochondrial protein HSP60, putative	2.8139	<0.0001
AFLA_012200	B8NYQ1	Hsp70 chaperone (HscA), putative	2.4216	<0.0001
AFLA_034050	B8N211	Aconitate hydratase, mitochondrial	2.2716	<0.0001
AFLA_028910	B8N3I8	Polyadenylate-binding protein	2.2287	0.0001
AFLA_040120	B8NAG7	Flavohemoprotein	2.2098	0.0002
AFLA_035620	B8N4E9	Hsp70 chaperone BiP/Kar2, putative	2.1612	<0.0001
AFLA_044620	B8NB87	Mitochondrial Hsp70 chaperone (Ssc70), putative	2.1373	<0.0001
AFLA_022380	B8N5B0	Molecular chaperone Hsp70	2.1337	0.0016
AFLA_093280	B8NKS4	Disulfide isomerase (TigA), putative	2.1103	0.0001
AFLA_033100	B8N3Y7	Phosphatidylinositol transporter, putative	1.8955	<0.0001
AFLA_119660	B8NWC9	ATP synthase subunit beta	1.7780	0.0026
AFLA_038700	A0A0D9NC88	Peptide hydrolase	1.7118	<0.0001
AFLA_008310	A0A0D9MUN9	Thiolase N-terminal domain protein	1.6783	<0.0001
AFLA_122720	A0A0D9MR79	Variant SH3 domain protein	1.6093	0.0008
AFLA_023500	A0A0D9N0I0	Ser-Thr-rich glycosyl-phosphatidyl-inositol-anchored membrane	1.3722	0.0011
AFLA_027810	A0A0D9MQC0	Subtilase family protein	1.2614	<0.0001
AFLA_095660	B8NKY3	Immunoglobulin G-binding protein H, putative	1.1468	<0.0001
AFLA_136640	B8NGN7	Translation elongation factor EF-2 subunit, putative	0.9712	0.0068
AFLA_108790	B8N8T4	Aldehyde dehydrogenase AldA, putative	0.6861	<0.0001
AFLA_112120	B8N9R6	Pyruvate carboxylase	0.6604	0.0026
AFLA_045980	A0A0D9N070	Prolyl oligopeptidase family protein	0.4090	<0.0001
		**NRRL3357 0 v 20mM**		
AFLA_105920	P41749	Endopolygalacturonase A	13.8028	<0.0001
AFLA_112130	A0A0D9MS17	Hydrophobic surface binding protein A	8.9999	<0.0001
AFLA_028800	B8N3H7	Eukaryotic translation initiation factor 5A	8.8726	<0.0001
AFLA_089380	A0A0D9MZ41	Endo-chitosanase	8.5050	<0.0001
AFLA_045750	B8NBK0	Antigenic mitochondrial protein HSP60, putative	8.0881	<0.0001

Table S2. Cont.

Gene ID	Uniprot ID	Annotation	Fold Change	*q* -value
AFLA_069370	B8NIQ9	Phosphoglycerate kinase	7.5484	<0.0001
AFLA_057240	A0A0D9MQY5	RNA recognition motif	7.0799	<0.0001
AFLA_019230	B8N6F9	Mismatched base pair and cruciform DNA recognition protein	7.0537	<0.0001
AFLA_093220	B8NKR8	Ran-specific GTPase-activating protein 1, putative	6.9233	<0.0001
AFLA_025760	B8N351	50S ribosomal protein L12	6.8469	0.0015
AFLA_136570	B8NGN0	Cytochrome c	6.8061	<0.0001
AFLA_043730	B8NAZ8	Nuclear protein export protein Yrb2, putative	6.3921	0.0002
AFLA_048510	B8NSI8	UV excision repair protein (RadW), putative	6.3291	0.0006
AFLA_066270	B8NID1	RNA-binding La domain protein	5.9871	<0.0001
AFLA_031780	B8N3U4	Telomere and ribosome associated protein Stm1, putative	5.9799	<0.0001
AFLA_091060	B8NLH9	Allergen Asp F3	5.7723	<0.0001
AFLA_003440	B8NQW0	Translation initiation factor 4B	5.7504	<0.0001
AFLA_044620	B8NB87	Mitochondrial Hsp70 chaperone (Ssc70), putative	5.5549	<0.0001
AFLA_030860	A0A0D9MYG7	Uncharacterized protein	5.4935	<0.0001
AFLA_106310	B8N837	Chaperonin, putative	5.4231	<0.0001
AFLA_091990	B8NKN3	Peptidyl-prolyl cis-trans isomerase	5.1617	<0.0001
AFLA_076710	A0A0D9N180	Malate dehydrogenase	4.8412	<0.0001
AFLA_028910	B8N3I8	Polyadenylate-binding protein	4.8204	<0.0001
AFLA_033100	B8N3Y7	Phosphatidylinositol transporter, putative	4.8118	<0.0001
AFLA_126870	B8NMS4	Uncharacterized protein	4.7704	0.0003
AFLA_132540	B8NH30	Thioredoxin	4.5661	<0.0001
AFLA_089270	A0A0D9N5A3	Basic region leucine zipper	4.3982	0.0004
AFLA_082510	B8MYW2	TCTP family protein	4.3492	0.0001
AFLA_091270	B8NKG1	Fumarate hydratase, putative	4.3014	<0.0001
AFLA_093660	B8NKW2	Solid-state culture expressed protein (Aos23), putative	4.2034	<0.0001
AFLA_112910	B8N9Z5	Uncharacterized protein	4.0491	0.0001
AFLA_133920	B8NFW3	Acyl CoA binding protein family	3.9799	<0.0001
AFLA_111100	B8N9G4	Mitochondrial peroxiredoxin Prx1, putative	3.8786	<0.0001
AFLA_006520	A0A0D9N6X4	Scd6-like Sm domain protein	3.8574	0.0016
AFLA_135540	B8NHE9	ATP synthase oligomycin sensitivity conferral protein, putative	3.8065	<0.0001
AFLA_094630	B8NM07	Triosephosphate isomerase	3.7901	<0.0001
AFLA_022470	B8N5B9	Adenylate kinase	3.7778	<0.0001
AFLA_043390	B8NAW4	Hsp70 chaperone (BiP), putative	3.7297	<0.0001
AFLA_012200	B8NYQ1	Hsp70 chaperone (HscA), putative	3.7234	<0.0001
AFLA_006300	B8NQF0	Nucleoside diphosphate kinase	3.6605	<0.0001
AFLA_035620	B8N4E9	Hsp70 chaperone BiP/Kar2, putative	3.6524	<0.0001
AFLA_004950	B8NQA4	Cytochrome c oxidase subunit Va, putative	3.5150	<0.0001
AFLA_035510	A0A0D9N8A2	KH domain protein	3.5028	<0.0001
AFLA_073480	B8MW01	Tropomyosin, putative	3.4259	0.0003
AFLA_057670	B8NE36	Neutral protease 2	3.3915	0.0002
AFLA_071010	B8NK52	Heat shock protein (Sti1), putative	3.3882	<0.0001
AFLA_023500	A0A0D9N0I0	Ser-Thr-rich glycosyl-phosphatidyl-inositol-anchored membrane	3.3598	0.0005
AFLA_029390	B8N174	HMG box protein, putative	3.3548	0.0007
AFLA_099000	Q8NK14	Cu,Zn-superoxide dismutase (Fragment)	3.2952	0.0012
AFLA_037490	B8N2H4	Eukaryotic translation initiation factor 3 subunit J	3.2690	<0.0001

Table S2. Cont.

Gene ID	Uniprot ID	Annotation	Fold Change	*q* -value
AFLA_022380	B8N5B0	Molecular chaperone Hsp70	3.2121	<0.0001
AFLA_054750	B8ND04	Malate dehydrogenase, NAD-dependent	3.2016	<0.0001
AFLA_117850	B8NW38	RPEL repeat protein	3.1798	0.0024
AFLA_002560	B8NPV3	60S ribosomal protein L37a	3.1527	0.0015
AFLA_036640	B8N4G1	PH domain protein	3.1291	<0.0001
AFLA_021820	B8N7G0	Nuclear movement protein NudC	3.0902	<0.0001
AFLA_007700	B8NXF1	Uncharacterized protein	3.0378	<0.0001
AFLA_025990	B8N0R4	Clathrin light chain	3.0098	0.0003
AFLA_051770	B8NSY4	Thioredoxin reductase	3.0022	<0.0001
AFLA_087950	B8MWA5	Isocitrate dehydrogenase LysB	2.9508	<0.0001
AFLA_067940	B8NIK7	Uncharacterized protein	2.9382	0.0002
AFLA_139300	Q5VD95	Ver-1	2.9378	<0.0001
AFLA_056260	B8ND65	Nascent polypeptide-associated complex (NAC) subunit, putative	2.8759	0.0003
AFLA_076450	B8MX58	Electron transfer flavoprotein alpha subunit, putative	2.8451	0.0004
AFLA_041260	B8NCF1	Ribosome associated DnaJ chaperone Zuotin, putative	2.8222	<0.0001
AFLA_086710	B8N031	Inorganic diphosphatase, putative	2.7256	<0.0001
AFLA_002670	B8NPW4	Curved DNA-binding protein (42 kDa protein)	2.6656	<0.0001
AFLA_032890	B8N1Y5	Uncharacterized protein	2.6651	<0.0001
AFLA_031960	B8N3W2	40S ribosomal protein S7e	2.5888	<0.0001
AFLA_012160	B8NYP6	Acetyl-CoA acetyltransferase, putative	2.5492	0.0001
AFLA_078380	B8MXQ1	Acetyl-coA hydrolase Ach1, putative	2.5228	<0.0001
AFLA_135200	B8NHB5	Uncharacterized protein	2.5227	0.0005
AFLA_069010	B8NJU1	S-adenosylmethionine synthase	2.4799	<0.0001
AFLA_128280	B8NP45	M protein repeat protein	2.4778	<0.0001
AFLA_056850	B8NDC4	Electron transfer flavoprotein, beta subunit, putative	2.4733	<0.0001
AFLA_122720	A0A0D9MR79	Variant SH3 domain protein	2.4677	<0.0001
AFLA_022270	B8N599	Uncharacterized protein	2.4341	<0.0001
AFLA_030180	B8N1F3	Protein mitochondrial targeting protein (Mas1), putative	2.4320	0.0004
AFLA_033620	B8N439	RNA binding protein, putative	2.4194	0.0007
AFLA_068840	B8NJS4	Aminomethyltransferase	2.3874	<0.0001
AFLA_113000	B8NA04	Integral ER membrane protein Scs2, putative	2.3700	0.0003
AFLA_014930	B8N6I0	Cofactor for methionyl-and glutamyl-tRNA synthetase, putative	2.3539	<0.0001
AFLA_091030	B8NLH6	Uncharacterized protein	2.3419	<0.0001
AFLA_061880	B8NEJ0	Pyridoxine biosynthesis protein	2.3126	<0.0001
AFLA_120630	B8NWM6	Glyoxylate/hydroxypyruvate reductase, putative	2.3036	<0.0001
N/A	A0A0D9MZ11	ATP synthase D chain mitochondrial ATP5H	2.2920	<0.0001
AFLA_080390	B8MYA2	6-phosphogluconolactonase, putative	2.2903	0.0006
AFLA_119660	B8NWC9	ATP synthase subunit beta	2.2785	<0.0001
AFLA_088570	B8MWG7	Uncharacterized protein	2.2651	0.0015
AFLA_093280	B8NKS4	Disulfide isomerase (TigA), putative	2.2141	<0.0001
AFLA_131280	B8NFM7	Actin cortical patch assembly protein Pan1, putative	2.1482	<0.0001
AFLA_048610	B8NSJ8	Succinyl-CoA synthetase alpha subunit, putative	2.0954	<0.0001
AFLA_052400	B8NS09	Isocitrate lyase	2.0952	<0.0001
AFLA_026470	B8N0W2	Aspartate aminotransferase	2.0949	<0.0001
AFLA_095570	B8NMC5	RNA annealing protein Yra1, putative	2.0178	0.0006

Table S2. Cont.

Gene ID	Uniprot ID	Annotation	Fold Change	*q* -value
AFLA_080930	B8MYF5	EF hand domain protein	1.9783	<0.0001
AFLA_007000	B8NQL8	Ubiquinol-cytochrome C reductase complex core protein 2	1.9670	0.0002
AFLA_060260	B8NEB7	Heat shock protein Hsp30/Hsp42, putative	1.9434	0.0001
AFLA_040120	B8NAG7	Flavohemoprotein	1.9342	<0.0001
AFLA_007020	B8NRD5	Citrate synthase	1.9242	<0.0001
AFLA_034050	B8N211	Aconitate hydratase, mitochondrial	1.8774	<0.0001
AFLA_025980	B8N0R3	Hsp90 co-chaperone Cdc37	1.8733	0.0008
AFLA_134120	B8NG73	Orotate phosphoribosyltransferase	1.8351	0.0031
AFLA_134340	B8NG94	BAR domain protein	1.8252	0.0006
AFLA_127390	B8NNC8	Proteasome regulatory particle subunit Rpt5, putative	1.8044	<0.0001
AFLA_042700	A0A0D9MTF9	RNA recognition motif	1.8035	0.0001
AFLA_099650	B8NTN4	Woronin body major protein, putative	1.7619	<0.0001
AFLA_029440	B8N179	NADH-cytochrome b5 reductase	1.7422	0.0003
AFLA_032870	B8N1Y3	cAMP-dependent protein kinase regulatory subunit	1.6347	0.0026
AFLA_022480	B8N5C0	26S proteasome regulatory particle subunit Rpn8, putative	1.6345	0.0018
AFLA_035540	B8N4E1	Signal transducing adapter molecule, putative	1.6233	0.0010
AFLA_045330	B8NBF8	Eukaryotic translation initiation factor subunit eIF-4F, putative	1.4860	0.0004
AFLA_029850	B8N1C0	Cysteine-rich secreted protein	0.9015	<0.0001
AFLA_097750	C5H8J2	SspA	0.7322	0.0007
AFLA_109320	B8N8Y6	3-hydroxyisobutyrate dehydrogenase	0.5787	0.0031
AFLA_106350	B8N841	ATP citrate lyase subunit (Acl), putatibe	0.5678	<0.0001
AFLA_044090	B8NB34	Uricase	0.5153	0.0034
AFLA_092640	B8NLP8	Alkaline phosphatase	0.5070	0.0014
AFLA_070990	B8NK50	UTP-glucose-1-phosphate uridylyltransferase Ugp1, putative	0.5061	0.0002
AFLA_110260	B8N980	Flavin-binding monooxygenase-like protein	0.5005	0.0007
AFLA_122180	B8NX31	Uncharacterized protein	0.4788	0.0024
AFLA_117760	B8NVT9	Phytase, putative	0.4773	<0.0001
AFLA_056170	B8NDX6	Catalase	0.4613	0.0002
AFLA_133990	B8NFX0	Secretory pathway gdp dissociation inhibitor	0.4509	<0.0001
AFLA_078650	B8MXS8	ATP synthase subunit alpha	0.4391	<0.0001
AFLA_124380	A0A0D9N439	Ricin-type beta-trefoil lectin domain-like protein	0.4300	0.0008
AFLA_020380	B8N500	Stomatin family protein	0.4267	0.0004
AFLA_069590	B8NIT1	Adenosylhomocysteinase	0.4243	0.0003
AFLA_079480	B8MY11	Oligopeptidase family protein	0.4130	0.0008
AFLA_050690	B8NRS8	Mitochondrial ADP,ATP carrier protein (Ant), putative	0.4082	0.0001
AFLA_044820	B8NBA7	Glucose-6-phosphate isomerase	0.4071	<0.0001
AFLA_031570	B8N3S3	Pyruvate decarboxylase PdcA, putative	0.4008	<0.0001
AFLA_083370	B8MZ48	Glutathione oxidoreductase Glr1, putative	0.3978	<0.0001
AFLA_036110	B8N2C6	4-hydroxyphenylpyruvate dioxygenase	0.3894	0.0004
AFLA_077860	B8MXJ9	Uncharacterized protein	0.3878	0.0006
AFLA_038790	B8NCH3	Adenosine deaminase family protein	0.3871	0.0004
AFLA_112120	B8N9R6	Pyruvate carboxylase	0.3819	<0.0001
AFLA_028260	B8N151	Probable glucan 1,3-beta-glucosidase A	0.3789	<0.0001
AFLA_060110	B8NEA2	Uncharacterized protein	0.3676	0.0001
AFLA_027070	B8N394	Acetyl-coenzyme A synthetase	0.3552	<0.0001

Table S2. Cont.

Gene ID	Uniprot ID	Annotation	Fold Change	*q*-value
AFLA_057770	B8NE46	Probable pectate lyase A	0.3498	<0.0001
AFLA_056350	B8ND74	2-methylcitrate dehydratase, putative	0.3473	0.0023
AFLA_001890	A0A0D9MRR2	GMC oxidoreductase	0.3396	0.0006
AFLA_034870	A0A0D9N8E0	Taurine catabolism dioxygenase TauD TfdA family protein	0.3376	<0.0001
AFLA_110600	B8N9B4	Aminopeptidase	0.3318	<0.0001
AFLA_078900	B8MXV3	Beta-hexosaminidase	0.3236	<0.0001
AFLA_128530	B8NP70	Delta-1-pyrroline-5-carboxylate dehydrogenase PrnC	0.3189	0.0005
AFLA_017100	A0A0D9MRY2	Beta-galactosidase	0.3185	<0.0001
AFLA_046730	B8NBU8	Uncharacterized protein	0.3156	0.0002
AFLA_125270	A0A0D9MSQ9	Uncharacterized protein	0.3106	<0.0001
AFLA_031760	B8N3U2	Glutaminase, putative	0.3012	0.0012
AFLA_039410	B8NCN4	Cell wall serine-threonine-rich galactomannoprotein Mp1	0.3002	<0.0001
AFLA_086900	B8N050	Alpha-mannosidase	0.2894	0.0004
AFLA_074520	B8MWJ5	Probable alpha-galactosidase A	0.2726	<0.0001
AFLA_110690	B8N9C3	Cytochrome c peroxidase Ccp1, putative	0.2689	<0.0001
AFLA_124500	B8NML4	Nitric oxide synthase, putative	0.2678	<0.0001
AFLA_036440	B8N2F9	Alanine aminotransferase, putative	0.2636	<0.0001
AFLA_101160	B8NUR3	40S ribosomal protein S9	0.2544	<0.0001
AFLA_036070	B8N2C2	Maleylacetoacetate isomerase MaiA	0.2498	0.0003
AFLA_008990	A0A0D9MP25	Carboxypeptidase	0.2484	<0.0001
AFLA_103940	B8NV99	Glutamate carboxypeptidase, putative	0.2475	<0.0001
AFLA_099220	B8NUG0	Uncharacterized protein	0.2439	0.0018
AFLA_122110	A0A0D9N4R3	Peroxidase	0.2412	<0.0001
AFLA_042090	B8NCX4	Fasciclin domain family protein	0.2401	<0.0001
AFLA_036840	B8N4I0	6-phosphogluconate dehydrogenase, decarboxylating	0.2376	<0.0001
AFLA_047350	B8NC10	Probable Xaa-Pro aminopeptidase pepP	0.2373	<0.0001
AFLA_112470	B8N9V1	Cobalamin-independent methionine synthase MetH/D	0.2364	<0.0001
AFLA_133150	B8NH91	Catechol dioxygenase, putative	0.2226	0.0008
AFLA_090690	B8NLE2	Mycelial catalase Cat1	0.2178	<0.0001
AFLA_130150	A0A0D9NA25	Uncharacterized protein	0.2168	<0.0001
AFLA_029310	B8N3M8	Alcohol dehydrogenase, zinc-containing, putative	0.2022	<0.0001
AFLA_139480	C9K205	Dimethylallyl tryptophan synthase	0.1989	<0.0001
AFLA_055060	B8ND35	NAD-dependent formate dehydrogenase AciA/Fdh	0.1975	<0.0001
AFLA_030140	B8N1E9	60S acidic ribosomal protein P0	0.1961	0.0003
AFLA_055230	B8ND52	Actin Act1	0.1924	<0.0001
AFLA_037960	B8N2M1	Glucosamine-fructose-6-phosphate aminotransferase	0.1894	<0.0001
AFLA_014520	B8N5V9	Multicopper oxidase, putative	0.1838	<0.0001
AFLA_038530	B8NC58	Extracellular metalloproteinase mep	0.1832	<0.0001
AFLA_101930	B8NU22	Succinate-semialdehyde dehydrogenase, putative	0.1668	<0.0001
AFLA_102010	B8NU30	Class V chitinase, putative	0.1642	<0.0001
AFLA_002090	B8NQR5	Extracellular serine carboxypeptidase, putative	0.1441	0.0007
AFLA_055450	B8NDQ4	Translation elongation factor eEF-1 subunit gamma, putative	0.1419	<0.0001
AFLA_025100	B8N2Y6	Glyceraldehyde-3-phosphate dehydrogenase	0.1397	<0.0001
AFLA_110160	B8N970	Probable dipeptidyl peptidase 4	0.1381	0.0007
AFLA_076180	B8MX31	Uncharacterized protein	0.1368	<0.0001

Table S2. Cont.

Gene ID	Uniprot ID	Annotation	Fold Change	*q* -value
AFLA_136640	B8NGN7	Translation elongation factor EF-2 subunit, putative	0.1365	<0.0001
AFLA_026140	B8N0S9	Alpha-amylase	0.1321	0.0051
AFLA_033400	A0A0D9N9J1	alpha-1,2-Mannosidase	0.1243	0.0008
AFLA_021870	A0A0D9MMH9	Glucanase	0.1221	<0.0001
AFLA_108100	B8N8L6	Argininosuccinate synthase	0.1211	<0.0001
AFLA_108790	B8N8T4	Aldehyde dehydrogenase AldA, putative	0.1165	<0.0001
AFLA_025510	B8N327	GPI anchored protein, putative	0.1109	<0.0001
AFLA_119040	B8NVX6	Muramidase, putative	0.1108	0.0002
AFLA_006960	B8NQL4	Molecular chaperone and allergen Mod-E/Hsp90/Hsp1	0.1079	<0.0001
AFLA_027810	A0A0D9MQC0	Subtilase family protein	0.0836	<0.0001
AFLA_051140	A0A0D9MWN4	Beta-glucosidase	0.0706	<0.0001
AFLA_139470	B8NI10	Beta-cyclopiazonate dehydrogenase	0.0613	<0.0001
AFLA_045980	A0A0D9N070	Prolyl oligopeptidase family protein	0.0585	<0.0001
AFLA_076170	B8MX30	Uncharacterized protein	0.0576	<0.0001
AFLA_070490	B8NK00	Uncharacterized protein	0.0511	<0.0001
AFLA_108410	B8N8P6	Dipeptidase	0.0441	<0.0001
AFLA_034640	B8N270	Peptide hydrolase	0.0418	<0.0001
		**K54A 0 v 10mM**		
AFLA_112130	A0A0D9MS17	Hydrophobic surface binding protein A	7.2344	<0.0001
AFLA_030880	B8N1M3	Uncharacterized protein	3.0305	0.0045
AFLA_003440	B8NQW0	Translation initiation factor 4B	2.8918	<0.0001
AFLA_007700	B8NXF1	Uncharacterized protein	2.7634	0.0002
AFLA_133920	B8NFW3	Acyl CoA binding protein family	2.2300	<0.0001
AFLA_031780	B8N3U4	Telomere and ribosome associated protein Stm1, putative	2.1348	0.0002
AFLA_057240	A0A0D9MQY5	RNA recognition motif	1.9996	<0.0001
AFLA_097190	B8NU47	Uncharacterized protein	1.9056	<0.0001
AFLA_039410	B8NCN4	Cell wall serine-threonine-rich galactomannoprotein Mp1	1.4781	<0.0001
AFLA_044620	B8NB87	Mitochondrial Hsp70 chaperone (Ssc70), putative	1.3370	<0.0001
AFLA_004950	B8NQA4	Cytochrome c oxidase subunit Va, putative	1.3313	0.0003
AFLA_071350	B8NJ18	UDP-N-acetylglucosamine pyrophosphorylase	0.6057	0.0003
AFLA_124500	B8NML4	Nitric oxide synthase, putative	0.5997	0.0007
AFLA_078650	B8MXS8	ATP synthase subunit alpha	0.5895	0.0002
AFLA_025100	B8N2Y6	Glyceraldehyde-3-phosphate dehydrogenase	0.5114	<0.0001
AFLA_042090	B8NCX4	Fasciclin domain family protein	0.5080	<0.0001
AFLA_122110	A0A0D9N4R3	Peroxidase	0.5028	0.0001
AFLA_101160	B8NUR3	40S ribosomal protein S9	0.5001	0.0012
AFLA_055060	B8ND35	NAD-dependent formate dehydrogenase AciA/Fdh	0.4053	<0.0001
AFLA_108410	B8N8P6	Dipeptidase	0.3654	<0.0001
AFLA_108790	B8N8T4	Aldehyde dehydrogenase AldA, putative	0.3300	<0.0001
AFLA_045980	A0A0D9N070	Prolyl oligopeptidase family protein	0.2662	<0.0001

Table S3. Biological function GO terms enriched among DEPs in *A. flavus* isolates in response to oxidative stress.

GO Term	Name	Result Freq.	$\log_{10} p$
**AF13 0 v 10mM**			
GO:0006457	protein folding	0.0090	-5.3444
GO:0005975	carbohydrate metabolic process	0.0526	-3.7034
GO:1901575	organic substance catabolic process	0.0461	-2.3419
GO:0009056	catabolic process	0.0482	-2.251
GO:0000387	spliceosomal snRNP assembly	0.0005	-2.152
GO:0000244	spliceosomal tri-snRNP complex assembly	0.0001	-2.152
GO:0006809	nitric oxide biosynthetic process	0.0002	-2.152
GO:1903409	reactive oxygen species biosynthetic process	0.0002	-2.152
GO:0042026	protein refolding	0.0007	-2.152
GO:0019427	acetyl-CoA biosynthetic process from acetate	0.0003	-2.152
GO:0019551	glutamate catabolic process to 2-oxoglutarate	0.0002	-2.152
GO:0043648	dicarboxylic acid metabolic process	0.0102	-2.107
GO:0032940	secretion by cell	0.0076	-2.107
GO:0046903	secretion	0.0081	-2.107
GO:0006403	RNA localization	0.0012	-2.0851
GO:0006979	response to oxidative stress	0.0058	-2.043
GO:1901071	glucosamine-containing compound metabolic process	0.0013	-2.043
GO:0007010	cytoskeleton organization	0.0079	-2.0076
GO:0034250	positive regulation of cellular amide metabolic process	0.0009	-1.9767
GO:0045727	positive regulation of translation	0.0008	-1.9767
GO:0006452	translational frameshifting	0.0002	-1.9767
GO:0043243	positive regulation of protein complex disassembly	0.0002	-1.9767
GO:0051017	actin filament bundle assembly	0.0005	-1.9767
GO:0051083	'de novo' cotranslational protein folding	0.0000	-1.9767
GO:0006106	fumarate metabolic process	0.0005	-1.9767
GO:0009414	response to water deprivation	0.0002	-1.9767
GO:0006100	(obsolete) tricarboxylic acid cycle intermediate metabolic process	0.0014	-1.9767
GO:0006022	aminoglycan metabolic process	0.0088	-1.965
GO:0006040	amino sugar metabolic process	0.0024	-1.965
GO:0008612	peptidyl-lysine modification to peptidyl-hypusine	0.0002	-1.8525
GO:0006458	'de novo' protein folding	0.0003	-1.8525
GO:0006972	hyperosmotic response	0.0001	-1.8525
GO:2001057	reactive nitrogen species metabolic process	0.0012	-1.8525
GO:0006108	malate metabolic process	0.0009	-1.7563
GO:0006103	2-oxoglutarate metabolic process	0.0002	-1.7563
GO:0001101	response to acid chemical	0.0012	-1.7563
GO:0008298	intracellular mRNA localization	0.0002	-1.7563
GO:0051130	positive regulation of cellular component organization	0.0037	-1.6779
GO:0030029	actin filament-based process	0.0040	-1.6129
GO:0006083	acetate metabolic process	0.0005	-1.6117
GO:0008150	biological_process	1.0000	-1.5823
GO:0044238	primary metabolic process	0.5374	-1.5713
GO:0022607	cellular component assembly	0.0248	-1.5436

Table S3. Cont.

GO Term	Name	Result Freq.	log_{10} *p*
GO:0071704	organic substance metabolic process	0.5836	-1.5154
GO:0030865	cortical cytoskeleton organization	0.0004	-1.504
GO:0044396	actin cortical patch organization	0.0001	-1.504
GO:0072593	reactive oxygen species metabolic process	0.0028	-1.504
GO:0009408	response to heat	0.0017	-1.504
GO:0043649	dicarboxylic acid catabolic process	0.0004	-1.459
GO:0010035	response to inorganic substance	0.0032	-1.459
GO:1901700	response to oxygen-containing compound	0.0050	-1.4183
GO:0051646	mitochondrion localization	0.0003	-1.4183
GO:0007155	cell adhesion	0.0054	-1.3158
GO:0022610	biological adhesion	0.0055	-1.3158
**AF13 0 v 25mM**			
GO:1901135	carbohydrate derivative metabolic process	0.0632	-2.5371
GO:0006048	UDP-N-acetylglucosamine biosynthetic process	0.0003	-2.5338
GO:0015980	energy derivation by oxidation of organic compounds	0.0118	-2.4489
GO:0006091	generation of precursor metabolites and energy	0.0194	-2.2363
GO:0019693	ribose phosphate metabolic process	0.0303	-2.2158
GO:0006012	galactose metabolic process	0.0011	-2.0578
GO:0044710	single-organism metabolic process	0.3168	-2.0319
GO:0009225	nucleotide-sugar metabolic process	0.0025	-1.971
GO:0090662	ATP hydrolysis coupled transmembrane transport	0.0100	-1.8092
GO:0015991	ATP hydrolysis coupled proton transport	0.0016	-1.8092
GO:0006113	fermentation	0.0002	-1.783
GO:0019682	glyceraldehyde-3-phosphate metabolic process	0.0044	-1.7351
GO:0051156	glucose 6-phosphate metabolic process	0.0032	-1.713
GO:0055086	nucleobase-containing small molecule metabolic process	0.0492	-1.6893
GO:0006739	NADP metabolic process	0.0036	-1.6529
GO:0044237	cellular metabolic process	0.5306	-1.5973
GO:0019637	organophosphate metabolic process	0.0615	-1.5464
GO:0044699	single-organism process	0.4657	-1.5314
GO:0009142	nucleoside triphosphate biosynthetic process	0.0065	-1.4979
GO:0006818	hydrogen transport	0.0115	-1.4851
GO:0006979	response to oxidative stress	0.0058	-1.4157
GO:0006950	response to stress	0.0458	-1.4069
GO:0006040	amino sugar metabolic process	0.0024	-1.375
GO:0006081	cellular aldehyde metabolic process	0.0075	-1.3206
GO:1901659	glycosyl compound biosynthetic process	0.0147	-1.3206
**NRRL3357 0 v 10mM**			
GO:0006457	protein folding	0.0090	-7.445
GO:0006616	SRP-dependent cotranslational protein targeting to membrane, translocati	0.0001	-3.6819
GO:0006094	gluconeogenesis	0.0026	-3.0022
GO:0033036	macromolecule localization	0.0303	-2.9823
GO:0006628	(obsolete) mitochondrial translocation	0.0014	-2.8592
GO:0070972	protein localization to endoplasmic reticulum	0.0019	-2.8592
GO:0071806	protein transmembrane transport	0.0053	-2.7758

Table S3. Cont.

GO Term	Name	Result Freq.	log_{10} *p*
GO:0065002	intracellular protein transmembrane transport	0.0028	-2.7758
GO:0006099	tricarboxylic acid cycle	0.0047	-2.6305
GO:0072350	tricarboxylic acid metabolic process	0.0050	-2.5663
GO:0051641	cellular localization	0.0204	-2.5491
GO:0045454	cell redox homeostasis	0.0086	-2.5068
GO:0006091	generation of precursor metabolites and energy	0.0194	-2.3309
GO:0072521	purine-containing compound metabolic process	0.0267	-2.3211
GO:0043648	dicarboxylic acid metabolic process	0.0102	-2.3043
GO:0042026	protein refolding	0.0007	-2.2522
GO:0007571	age-dependent general metabolic decline	0.0001	-2.2522
GO:0001321	age-dependent general metabolic decline involved in replicative cell aging	0.0000	-2.2522
GO:0006100	(obsolete) tricarboxylic acid cycle intermediate metabolic process	0.0014	-2.0767
GO:0006740	NADPH regeneration	0.0001	-2.0767
GO:0071702	organic substance transport	0.0498	-2.0668
GO:0016051	carbohydrate biosynthetic process	0.0108	-2.0074
GO:0044283	small molecule biosynthetic process	0.0568	-1.9953
GO:0044281	small molecule metabolic process	0.1514	-1.9756
GO:0000741	karyogamy	0.0001	-1.9524
GO:0009117	nucleotide metabolic process	0.0417	-1.9504
GO:0008150	biological_process	1.0000	-1.9349
GO:0072522	purine-containing compound biosynthetic process	0.0150	-1.8789
GO:0006108	malate metabolic process	0.0009	-1.856
GO:0061024	membrane organization	0.0076	-1.7462
GO:0072524	pyridine-containing compound metabolic process	0.0135	-1.7352
GO:0006733	oxidoreduction coenzyme metabolic process	0.0127	-1.6824
GO:0019541	propionate metabolic process	0.0003	-1.6536
GO:1901566	organonitrogen compound biosynthetic process	0.1406	-1.6262
GO:0016482	cytosolic transport	0.0008	-1.6048
GO:0044396	actin cortical patch organization	0.0001	-1.6031
GO:0006446	regulation of translational initiation	0.0009	-1.6031
GO:0030866	cortical actin cytoskeleton organization	0.0003	-1.6031
GO:0046459	short-chain fatty acid metabolic process	0.0003	-1.6031
GO:0030865	cortical cytoskeleton organization	0.0004	-1.6031
GO:0055086	nucleobase-containing small molecule metabolic process	0.0492	-1.566
GO:0006986	response to unfolded protein	0.0004	-1.5579
GO:0009113	purine nucleobase biosynthetic process	0.0012	-1.5579
GO:0035966	response to topologically incorrect protein	0.0005	-1.5171
GO:0044711	single-organism biosynthetic process	0.1086	-1.5168
GO:0015914	phospholipid transport	0.0008	-1.4799
GO:1901564	organonitrogen compound metabolic process	0.1789	-1.4506
GO:0007568	aging	0.0009	-1.4457
GO:0019538	protein metabolic process	0.1849	-1.4162
GO:0034308	primary alcohol metabolic process	0.0005	-1.3847
GO:0006537	glutamate biosynthetic process	0.0009	-1.3847
GO:0006067	ethanol metabolic process	0.0002	-1.3847

Table S3. Cont.

GO Term	Name	Result Freq.	$\log_{10} p$
GO:0019637	organophosphate metabolic process	0.0615	-1.3732
GO:0090662	ATP hydrolysis coupled transmembrane transport	0.0100	-1.3573
GO:0015991	ATP hydrolysis coupled proton transport	0.0016	-1.3573
GO:0006414	translational elongation	0.0078	-1.3573
GO:0006508	proteolysis	0.0522	-1.3097
GO:0006893	Golgi to plasma membrane transport	0.0003	-1.3073
	**NRRL3357 0 v 20mM**		
GO:1901564	organonitrogen compound metabolic process	0.1789	-9.6479
GO:0044281	small molecule metabolic process	0.1514	-9.0182
GO:0071704	organic substance metabolic process	0.5836	-7.7614
GO:0006979	response to oxidative stress	0.0058	-7.4622
GO:0006457	protein folding	0.0090	-7.3287
GO:0043648	dicarboxylic acid metabolic process	0.0102	-6.6855
GO:0006082	organic acid metabolic process	0.0909	-6.2381
GO:0044238	primary metabolic process	0.5374	-6.1893
GO:0055086	nucleobase-containing small molecule metabolic process	0.0492	-5.5771
GO:0072350	tricarboxylic acid metabolic process	0.0050	-5.4918
GO:0006732	coenzyme metabolic process	0.0311	-5.3998
GO:0072524	pyridine-containing compound metabolic process	0.0135	-5.2466
GO:0019318	hexose metabolic process	0.0064	-4.9098
GO:0006091	generation of precursor metabolites and energy	0.0194	-4.8957
GO:0072521	purine-containing compound metabolic process	0.0267	-4.8621
GO:0009117	nucleotide metabolic process	0.0417	-4.838
GO:0009405	pathogenesis	0.0010	-4.7576
GO:0006536	glutamate metabolic process	0.0020	-4.7089
GO:1901605	alpha-amino acid metabolic process	0.0363	-4.7038
GO:0051186	cofactor metabolic process	0.0399	-4.6503
GO:0006099	tricarboxylic acid cycle	0.0047	-4.6192
GO:1901566	organonitrogen compound biosynthetic process	0.1406	-4.3131
GO:0044710	single-organism metabolic process	0.3168	-4.3005
GO:0045454	cell redox homeostasis	0.0086	-4.2931
GO:0005975	carbohydrate metabolic process	0.0526	-4.2293
GO:1901575	organic substance catabolic process	0.0461	-4.1537
GO:0044237	cellular metabolic process	0.5306	-4.0034
GO:0006807	nitrogen compound metabolic process	0.3874	-3.9359
GO:0009056	catabolic process	0.0482	-3.8822
GO:1901135	carbohydrate derivative metabolic process	0.0632	-3.8544
GO:0006508	proteolysis	0.0522	-3.769
GO:1901657	glycosyl compound metabolic process	0.0296	-3.7326
GO:0006950	response to stress	0.0458	-3.6649
GO:0019637	organophosphate metabolic process	0.0615	-3.5688
GO:0019538	protein metabolic process	0.1849	-3.5413
GO:0008152	metabolic process	0.7539	-3.4523
GO:0006518	peptide metabolic process	0.0596	-3.3196
GO:0006414	translational elongation	0.0078	-3.3164

Table S3. Cont.

GO Term	Name	Result Freq.	$\log_{10} p$
GO:0006083	acetate metabolic process	0.0005	-3.2122
GO:0044711	single-organism biosynthetic process	0.1086	-3.1117
GO:0006081	cellular aldehyde metabolic process	0.0075	-3.1109
GO:0019682	glyceraldehyde-3-phosphate metabolic process	0.0044	-3.061
GO:0006790	sulfur compound metabolic process	0.0182	-3.0298
GO:0033036	macromolecule localization	0.0303	-3.0086
GO:0051156	glucose 6-phosphate metabolic process	0.0032	-2.9848
GO:0072593	reactive oxygen species metabolic process	0.0028	-2.9319
GO:0006793	phosphorus metabolic process	0.1351	-2.9036
GO:0006403	RNA localization	0.0012	-2.8409
GO:0043649	dicarboxylic acid catabolic process	0.0004	-2.8134
GO:0043603	cellular amide metabolic process	0.0688	-2.7522
GO:0006730	one-carbon metabolic process	0.0033	-2.7059
GO:0044283	small molecule biosynthetic process	0.0568	-2.6886
GO:0006100	(obsolete) tricarboxylic acid cycle intermediate metabolic process	0.0014	-2.6293
GO:0006106	fumarate metabolic process	0.0005	-2.6293
GO:0055114	oxidation-reduction process	0.1506	-2.6247
GO:0034248	regulation of cellular amide metabolic process	0.0070	-2.5971
GO:0006417	regulation of translation	0.0069	-2.5971
GO:0008150	biological_process	1.0000	-2.5961
GO:0010608	posttranscriptional regulation of gene expression	0.0072	-2.4885
GO:0009066	aspartate family amino acid metabolic process	0.0083	-2.4628
GO:0009987	cellular process	0.6378	-2.4605
GO:0051704	multi-organism process	0.0075	-2.4575
GO:0065008	regulation of biological quality	0.0340	-2.4073
GO:0032787	monocarboxylic acid metabolic process	0.0249	-2.3906
GO:0006637	acyl-CoA metabolic process	0.0025	-2.3554
GO:0035383	thioester metabolic process	0.0025	-2.3554
GO:0006537	glutamate biosynthetic process	0.0009	-2.3554
GO:0044723	single-organism carbohydrate metabolic process	0.0246	-2.3085
GO:0000011	vacuole inheritance	0.0001	-2.2826
GO:0051246	regulation of protein metabolic process	0.0155	-2.2478
GO:0008298	intracellular mRNA localization	0.0002	-2.2429
GO:0006108	malate metabolic process	0.0009	-2.2429
GO:0006090	pyruvate metabolic process	0.0082	-2.1654
GO:0051641	cellular localization	0.0204	-2.1066
GO:0007039	protein catabolic process in the vacuole	0.0000	-2.1026
GO:0006572	tyrosine catabolic process	0.0001	-2.1026
GO:0006570	tyrosine metabolic process	0.0006	-1.9834
GO:0006616	SRP-dependent cotranslational protein targeting to membrane, translocati	0.0001	-1.9834
GO:0070727	cellular macromolecule localization	0.0156	-1.9718
GO:0009058	biosynthetic process	0.3161	-1.9693
GO:1901576	organic substance biosynthetic process	0.3037	-1.9483
GO:0034641	cellular nitrogen compound metabolic process	0.3414	-1.9398
GO:0048308	organelle inheritance	0.0003	-1.9263

Table S3. Cont.

GO Term	Name	Result Freq.	$\log_{10} p$
GO:0006801	superoxide metabolic process	0.0014	-1.88
GO:0019541	propionate metabolic process	0.0003	-1.88
GO:0044763	single-organism cellular process	0.2754	-1.7941
GO:0044396	actin cortical patch organization	0.0001	-1.7888
GO:0030866	cortical actin cytoskeleton organization	0.0003	-1.7888
GO:1902222	erythrose 4-phosphate/phosphoenolpyruvate family amino acid catabolic p	0.0003	-1.7888
GO:0046459	short-chain fatty acid metabolic process	0.0003	-1.7888
GO:0030865	cortical cytoskeleton organization	0.0004	-1.7888
GO:0050896	response to stimulus	0.1221	-1.7364
GO:0010035	response to inorganic substance	0.0032	-1.7073
GO:1902221	erythrose 4-phosphate/phosphoenolpyruvate family amino acid metabolic	0.0008	-1.7073
GO:0006986	response to unfolded protein	0.0004	-1.7073
GO:0072528	pyrimidine-containing compound biosynthetic process	0.0090	-1.7015
GO:0044267	cellular protein metabolic process	0.1429	-1.6907
GO:0035966	response to topologically incorrect protein	0.0005	-1.6339
GO:0051646	mitochondrion localization	0.0003	-1.6339
GO:1902600	hydrogen ion transmembrane transport	0.0102	-1.6242
GO:0016192	vesicle-mediated transport	0.0109	-1.5817
GO:0015914	phospholipid transport	0.0008	-1.567
GO:0072527	pyrimidine-containing compound metabolic process	0.0104	-1.5526
GO:0007033	vacuole organization	0.0010	-1.5188
GO:0044249	cellular biosynthetic process	0.3005	-1.5094
GO:0006818	hydrogen transport	0.0115	-1.4547
GO:0009112	nucleobase metabolic process	0.0048	-1.4547
GO:0042026	protein refolding	0.0007	-1.4
GO:0001887	selenium compound metabolic process	0.0001	-1.4
GO:0007571	age-dependent general metabolic decline	0.0001	-1.4
GO:0000052	citrulline metabolic process	0.0002	-1.4
GO:0016259	selenocysteine metabolic process	0.0004	-1.4
GO:0006011	UDP-glucose metabolic process	0.0005	-1.4
GO:0050821	protein stabilization	0.0005	-1.4
GO:0000387	spliceosomal snRNP assembly	0.0005	-1.4
GO:0031647	regulation of protein stability	0.0007	-1.4
GO:0046039	GTP metabolic process	0.0010	-1.4
GO:0000053	argininosuccinate metabolic process	0.0000	-1.4
GO:0001321	age-dependent general metabolic decline involved in replicative cell aging	0.0000	-1.4
GO:0000244	spliceosomal tri-snRNP complex assembly	0.0001	-1.4
GO:0034308	primary alcohol metabolic process	0.0005	-1.397
GO:0006067	ethanol metabolic process	0.0002	-1.397
GO:0022607	cellular component assembly	0.0248	-1.3685
GO:0046903	secretion	0.0081	-1.3663
GO:0090662	ATP hydrolysis coupled transmembrane transport	0.0100	-1.3483
GO:0044271	cellular nitrogen compound biosynthetic process	0.2250	-1.3437
GO:0006575	cellular modified amino acid metabolic process	0.0079	-1.3226
GO:0051168	nuclear export	0.0012	-1.3119

Table S3. Cont.

GO Term	Name	Result Freq.	$\log_{10} p$
	**K54A 0 v 10mM**		
GO:1903409	reactive oxygen species biosynthetic process	0.0002	-2.3583
GO:0006809	nitric oxide biosynthetic process	0.0002	-2.3583
GO:0009117	nucleotide metabolic process	0.0417	-2.2536
GO:0006048	UDP-N-acetylglucosamine biosynthetic process	0.0003	-2.1826
GO:0015942	formate metabolic process	0.0007	-2.1826
GO:0042183	formate catabolic process	0.0001	-2.1826
GO:0006735	NADH regeneration	0.0000	-2.1826
GO:2001057	reactive nitrogen species metabolic process	0.0012	-2.0581
GO:0072524	pyridine-containing compound metabolic process	0.0135	-1.9404
GO:0006733	oxidoreduction coenzyme metabolic process	0.0127	-1.8867
GO:0006450	regulation of translational fidelity	0.0030	-1.883
GO:0055086	nucleobase-containing small molecule metabolic process	0.0492	-1.8563
GO:0006083	acetate metabolic process	0.0005	-1.8165
GO:0006734	NADH metabolic process	0.0001	-1.8165
GO:0072593	reactive oxygen species metabolic process	0.0028	-1.7082
GO:0019637	organophosphate metabolic process	0.0615	-1.6548
GO:0009225	nucleotide-sugar metabolic process	0.0025	-1.622
GO:0055114	oxidation-reduction process	0.1506	-1.6206
GO:0006091	generation of precursor metabolites and energy	0.0194	-1.5419
GO:0072521	purine-containing compound metabolic process	0.0267	-1.5353
GO:0007155	cell adhesion	0.0054	-1.5186
GO:0022610	biological adhesion	0.0055	-1.5186
GO:1901135	carbohydrate derivative metabolic process	0.0632	-1.5012
GO:0034308	primary alcohol metabolic process	0.0005	-1.4891
GO:0006067	ethanol metabolic process	0.0002	-1.4891
GO:0090662	ATP hydrolysis coupled transmembrane transport	0.0100	-1.4615
GO:0006094	gluconeogenesis	0.0026	-1.4615
GO:0015991	ATP hydrolysis coupled proton transport	0.0016	-1.4615
GO:1901657	glycosyl compound metabolic process	0.0296	-1.4376
GO:0034641	cellular nitrogen compound metabolic process	0.3414	-1.4263
GO:0006628	(obsolete) mitochondrial translocation	0.0014	-1.3882
GO:0060249	anatomical structure homeostasis	0.0019	-1.3664
GO:0000723	telomere maintenance	0.0013	-1.3664
GO:0032200	telomere organization	0.0013	-1.3664

Table S4. Cell localization GO terms enriched among DEPs in *A. flavus* isolates in response to oxidative stress.

GO Term	Name	Result Freq.	$\log_{10} p$
	**AF13 0 v 10mM**		
GO:0005737	cytoplasm	0.2602	-2.1202
GO:0005854	nascent polypeptide-associated complex	0.0000	-1.9767
GO:0000783	nuclear telomere cap complex	0.0001	-1.9767
GO:0005823	central plaque of spindle pole body	0.0000	-1.9767
GO:0009277	fungal-type cell wall	0.0003	-1.7835
GO:0000131	incipient cellular bud site	0.0002	-1.7563
GO:0005618	cell wall	0.0029	-1.6869
GO:0030312	external encapsulating structure	0.0092	-1.6013
GO:0008541	proteasome regulatory particle, lid subcomplex	0.0002	-1.5544
GO:0005788	endoplasmic reticulum lumen	0.0003	-1.5544
GO:0044444	cytoplasmic part	0.1266	-1.4616
GO:0005934	cellular bud tip	0.0002	-1.4590
GO:0044450	microtubule organizing center part	0.0008	-1.4590
GO:0005937	mating projection	0.0004	-1.3472
GO:0042995	cell projection	0.0105	-1.3472
	**AF13 0 v 25mM**		
GO:0044429	mitochondrial part	0.0120	-2.8686
GO:0005754	mitochondrial proton-transporting ATP synthase, catalytic core	0.0001	-2.5338
GO:0045267	proton-transporting ATP synthase, catalytic core	0.0001	-2.5338
GO:0005737	cytoplasm	0.2602	-2.3019
GO:0045277	respiratory chain complex IV	0.0005	-2.2333
GO:0098796	membrane protein complex	0.0247	-2.1446
GO:0005739	mitochondrion	0.0216	-2.1286
GO:0070069	cytochrome complex	0.0010	-1.8988
GO:0070469	respiratory chain	0.0030	-1.7830
GO:0031967	organelle envelope	0.0126	-1.7779
GO:0031975	envelope	0.0232	-1.7548
GO:0005625	(obsolete) soluble fraction	0.0014	-1.5533
GO:0016469	proton-transporting two-sector ATPase complex	0.0066	-1.5247
GO:0031090	organelle membrane	0.0200	-1.3787
	**NRRL3357 0 v 10mM**		
GO:0044444	cytoplasmic part	0.1266	-10.0571
GO:0005737	cytoplasm	0.2602	-8.8752
GO:0005788	endoplasmic reticulum lumen	0.0003	-5.6430
GO:0044424	intracellular part	0.3565	-4.9227
GO:0005622	intracellular	0.4118	-4.6872
GO:0044464	cell part	0.5239	-3.9975
GO:0005623	cell	0.5355	-3.9799
GO:0005829	cytosol	0.0255	-3.6767
GO:0005575	cellular_component	1.0000	-3.2506
GO:0005739	mitochondrion	0.0216	-2.9146
GO:0044432	endoplasmic reticulum part	0.0082	-2.6485
GO:0044422	organelle part	0.0943	-2.4058

Table S4. Cont.

GO Term	Name	Result Freq.	$\log_{10} p$
GO:0044429	mitochondrial part	0.0120	-2.3397
GO:0043226	organelle	0.2079	-2.1510
GO:0043229	intracellular organelle	0.1992	-2.1510
GO:0070013	intracellular organelle lumen	0.0274	-2.1334
GO:0005754	mitochondrial proton-transporting ATP synthase, catalytic core	0.0001	-2.0767
GO:0045267	proton-transporting ATP synthase, catalytic core	0.0001	-2.0767
GO:0031974	membrane-enclosed lumen	0.0274	-2.0253
GO:0031090	organelle membrane	0.0200	-1.8333
GO:0030132	clathrin coat of coated pit	0.0004	-1.7111
GO:0005844	polysome	0.0004	-1.6536
GO:0098589	membrane region	0.0012	-1.6031
GO:0043231	intracellular membrane-bounded organelle	0.1376	-1.5814
GO:0012505	endomembrane system	0.0281	-1.5762
GO:0030529	intracellular ribonucleoprotein complex	0.0529	-1.3848
GO:0030658	transport vesicle membrane	0.0010	-1.3847
**NRRL3357 0 v 20mM**			
GO:0005737	cytoplasm	0.2602	-21.6660
GO:0044444	cytoplasmic part	0.1266	-18.9073
GO:0005829	cytosol	0.0255	-11.7075
GO:0005739	mitochondrion	0.0216	-10.1688
GO:0044424	intracellular part	0.3565	-9.7485
GO:0005622	intracellular	0.4118	-8.8318
GO:0044464	cell part	0.5239	-7.3714
GO:0005623	cell	0.5355	-7.3042
GO:0044429	mitochondrial part	0.0120	-6.5324
GO:0031970	organelle envelope lumen	0.0009	-5.3095
GO:0005758	mitochondrial intermembrane space	0.0008	-5.3095
GO:0031967	organelle envelope	0.0126	-4.3752
GO:0031975	envelope	0.0232	-4.2429
GO:0005575	cellular_component	1.0000	-4.1326
GO:0044422	organelle part	0.0943	-3.9772
GO:0044446	intracellular organelle part	0.0894	-3.9772
GO:0005840	ribosome	0.0420	-3.1087
GO:0032991	macromolecular complex	0.1401	-2.9354
GO:0031974	membrane-enclosed lumen	0.0274	-2.6580
GO:0045267	proton-transporting ATP synthase, catalytic core	0.0001	-2.6293
GO:0043226	organelle	0.2079	-2.6264
GO:0043229	intracellular organelle	0.1992	-2.6264
GO:0045259	proton-transporting ATP synthase complex	0.0049	-2.4333
GO:0009277	fungal-type cell wall	0.0003	-2.1899
GO:0005618	cell wall	0.0029	-1.9869
GO:0030132	clathrin coat of coated pit	0.0004	-1.9834
GO:0005773	vacuole	0.0046	-1.9631
GO:0000323	lytic vacuole	0.0026	-1.9406
GO:0031090	organelle membrane	0.0200	-1.8981

Table S4. Cont.

GO Term	Name	Result Freq.	$\log_{10} p$
GO:0005844	polysome	0.0004	-1.8800
GO:0005788	endoplasmic reticulum lumen	0.0003	-1.8800
GO:0030312	external encapsulating structure	0.0092	-1.8099
GO:0098589	membrane region	0.0012	-1.7888
GO:0005576	extracellular region	0.0238	-1.6949
GO:0098796	membrane protein complex	0.0247	-1.6305
GO:0005625	(obsolete) soluble fraction	0.0014	-1.6242
GO:0016469	proton-transporting two-sector ATPase complex	0.0066	-1.5526
GO:0022626	cytosolic ribosome	0.0019	-1.5342
GO:0070069	cytochrome complex	0.0010	-1.5058
GO:0043228	non-membrane-bounded organelle	0.0841	-1.4612
GO:0044445	cytosolic part	0.0050	-1.4255
GO:0005952	cAMP-dependent protein kinase complex	0.0001	-1.4000
GO:0017102	methionyl glutamyl tRNA synthetase complex	0.0001	-1.4000
GO:0005853	eukaryotic translation elongation factor 1 complex	0.0001	-1.4000
GO:0030658	transport vesicle membrane	0.0010	-1.3970
GO:0070469	respiratory chain	0.0030	-1.3028
**K54A 0 v 10mM**			
GO:0005740	mitochondrial envelope	0.0090	-3.4427
GO:0044444	cytoplasmic part	0.1266	-3.0460
GO:0031975	envelope	0.0232	-2.9488
GO:0005737	cytoplasm	0.2602	-2.7850
GO:0005739	mitochondrion	0.0216	-2.6358
GO:0098796	membrane protein complex	0.0247	-2.5098
GO:0031090	organelle membrane	0.0200	-2.2217
GO:0000783	nuclear telomere cap complex	0.0001	-2.1826
GO:0045267	proton-transporting ATP synthase, catalytic core	0.0001	-2.1826
GO:0005754	mitochondrial proton-transporting ATP synthase, catalytic core	0.0001	-2.1826
GO:0005829	cytosol	0.0255	-2.0396
GO:0044422	organelle part	0.0943	-1.9536
GO:0044446	intracellular organelle part	0.0894	-1.9536
GO:0045277	respiratory chain complex IV	0.0005	-1.8830
GO:0030132	clathrin coat of coated pit	0.0004	-1.8165
GO:0098589	membrane region	0.0012	-1.7082
GO:0070069	cytochrome complex	0.0010	-1.5503
GO:0030658	transport vesicle membrane	0.0010	-1.4891
GO:0070469	respiratory chain	0.0030	-1.4356
GO:0032991	macromolecular complex	0.1401	-1.3546
GO:0043226	organelle	0.2079	-1.3263
GO:0043229	intracellular organelle	0.1992	-1.3263
GO:0098797	plasma membrane protein complex	0.0094	-1.3259

Table S5. KEGG pathway terms enriched among DEPs in *A. flavus* isolates in response to oxidative stress.

Pathway ID	Name	Result Count	p-value
	**AF13 0 v 10mM**		
ec00710	Carbon fixation in photosynthetic organisms	3	0.0001
ec00531	Glycosaminoglycan degradation	3	0.0009
ec00500	Starch and sucrose metabolism	3	0.0051
ec00513	Various types of N-glycan biosynthesis	2	0.0081
ec00630	Glyoxylate and dicarboxylate metabolism	2	0.0114
ec00620	Pyruvate metabolism	2	0.0166
ec00010	Glycolysis / Gluconeogenesis	2	0.0195
ec00430	Taurine and hypotaurine metabolism	1	0.0355
	**AF13 0 v 25mM**		
ec00520	Amino sugar and nucleotide sugar metabolism	2	0.0311
	**NRRL3357 0 v 10mM**		
ec00620	Pyruvate metabolism	4	<0.0001
ec00720	Carbon fixation pathways in prokaryotes	3	0.0013
ec00710	Carbon fixation in photosynthetic organisms	2	0.0013
ec00020	Citrate cycle (TCA cycle)	2	0.0016
ec00630	Glyoxylate and dicarboxylate metabolism	2	0.0032
ec00280	Valine, leucine and isoleucine degradation	2	0.0041
ec00010	Glycolysis / Gluconeogenesis	2	0.0056
ec00071	Fatty acid degradation	2	0.0097
ec00072	Synthesis and degradation of ketone bodies	1	0.0175
	**NRRL3357 0 v 20mM**		
ec00020	Citrate cycle (TCA cycle)	8	<0.0001
ec00630	Glyoxylate and dicarboxylate metabolism	9	<0.0001
ec00620	Pyruvate metabolism	9	<0.0001
ec00710	Carbon fixation in photosynthetic organisms	7	<0.0001
ec00010	Glycolysis / Gluconeogenesis	7	0.0003
ec00480	Glutathione metabolism	7	0.0005
ec00250	Alanine, aspartate and glutamate metabolism	6	0.0010
ec00720	Carbon fixation pathways in prokaryotes	9	0.0010
ec00500	Starch and sucrose metabolism	7	0.0047
ec00531	Glycosaminoglycan degradation	5	0.0050
ec00520	Amino sugar and nucleotide sugar metabolism	8	0.0249
ec00270	Cysteine and methionine metabolism	6	0.0403
ec00030	Pentose phosphate pathway	3	0.0435
	**K54A 0 v 10mM**		
ec00010	Glycolysis / Gluconeogenesis	2	0.0056

Table S6. Annotations of specific proteins in the protein-protein interaction analysis for NRRL3357.

ID	STRING ID	Annotation
AFLA_001890	CADAFLAP00010255	Cellobiose dehydrogenase
AFLA_002090	CADAFLAP00010275	Extracellular serine carboxypeptidase, putative
AFLA_002560	CADAFLAP00010322	60S ribosomal protein L37a
AFLA_002670	CADAFLAP00010333	Curved DNA-binding protein (42 kDa protein)
AFLA_003440	CADAFLAP00010410	Translation initiation factor 4B
AFLA_004950	CADAFLAP00010561	Cytochrome c oxidase subunit Va, putative
AFLA_006300	CADAFLAP00010695	Nucleoside diphosphate kinase (EC 2.7.4.6)
AFLA_006520	CADAFLAP00010715	CRAL/TRIO domain protein
AFLA_006960	CADAFLAP00010759	Molecular chaperone and allergen Mod-E/Hsp90/Hsp1
AFLA_007000	CADAFLAP00010763	Ubiquinol-cytochrome C reductase complex core protein 2
AFLA_007020	CADAFLAP00010765	Citrate synthase
AFLA_007700	CADAFLAP00012881	Putative uncharacterized protein
AFLA_008310	CADAFLAP00012942	Acetyl-CoA-acetyltransferase, putative
AFLA_008990	cpyA	Carboxypeptidase CpyA/Prc1, putative
AFLA_012160	CADAFLAP00013326	Acetyl-CoA acetyltransferase, putative
AFLA_012200	CADAFLAP00013330	Hsp70 chaperone (HscA), putative
AFLA_014520	CADAFLAP00003337	Multicopper oxidase, putative
AFLA_014930	CADAFLAP00003378	Cofactor for methionyl-and glutamyl-tRNA synthetase, putative
AFLA_017100	lacA	Beta-galactosidase (EC 3.2.1.23)
AFLA_019230	CADAFLAP00003808	Mismatched base pair and cruciform DNA recognition protein
AFLA_020380	CADAFLAP00003923	Stomatin family protein
AFLA_021820	CADAFLAP00004067	Nuclear movement protein NudC
AFLA_021870	cbhA	Cellobiohydrolase celD
AFLA_022270	CADAFLAP00004112	Putative uncharacterized protein
AFLA_022380	CADAFLAP00004123	Molecular chaperone Hsp70
AFLA_022470	ADK1	Adenylate kinase, putative
AFLA_022480	CADAFLAP00004133	26S proteasome regulatory particle subunit Rpn8, putative
AFLA_023500	CADAFLAP00001726	Extracellular conserved serine-rich protein
AFLA_025100	CADAFLAP00001886	Glyceraldehyde 3-phosphate dehydrogenase (EC 1.2.1.12)
AFLA_025510	CADAFLAP00001927	GPI anchored protein, putative
AFLA_025760	CADAFLAP00001951	50S ribosomal protein L12
AFLA_025980	CADAFLAP00001973	Hsp90 co-chaperone Cdc37
AFLA_025990	CADAFLAP00001974	Clathrin light chain
AFLA_026140	CADAFLAP00001989	Alpha-amylase, putative
AFLA_026470	CADAFLAP00002022	Aspartate aminotransferase (EC 2.6.1.1)
AFLA_027070	CADAFLAP00002082	Acetyl-coenzyme A synthetase FacA
AFLA_027810	alp	Alkaline protease
AFLA_028260	exgA	Exo-beta-1,3-glucanase (Exg1), putative
AFLA_028800	CADAFLAP00002255	Eukaryotic translation initiation factor eIF-5A
AFLA_028910	CADAFLAP00002266	Polyadenylate-binding protein
AFLA_029310	CADAFLAP00002306	Alcohol dehydrogenase, zinc-containing, putative
AFLA_029390	CADAFLAP00002314	HMG box protein, putative
AFLA_029440	CADAFLAP00002319	NADH-cytochrome b5 reductase, putative
AFLA_029850	CADAFLAP00002360	Cysteine-rich secreted protein

Table S6. Cont.

ID	STRING ID	Annotation
AFLA_030140	CADAFLAP00002389	60S ribosomal protein P0
AFLA_030180	CADAFLAP00002393	Protein mitochondrial targeting protein (Mas1), putative
AFLA_030860	CADAFLAP00002461	RNAPII degradation factor Def1, putative
AFLA_031570	CADAFLAP00002532	Pyruvate decarboxylase PdcA, putative
AFLA_031760	CADAFLAP00002551	Glutaminase, putative
AFLA_031780	CADAFLAP00002553	Telomere and ribosome associated protein Stm1, putative
AFLA_031960	CADAFLAP00002571	40S ribosomal protein S7e
AFLA_032870	CADAFLAP00002662	cAMP-dependent protein kinase regulatory subunit PkaR
AFLA_032890	CADAFLAP00002664	Putative uncharacterized protein
AFLA_033100	CADAFLAP00002685	Phosphatidylinositol transporter, putative
AFLA_033400	CADAFLAP00002715	Mannosidase MsdS
AFLA_033620	CADAFLAP00002737	RNA binding protein, putative
AFLA_034050	CADAFLAP00002780	Mitochondrial aconitate hydratase, putative
AFLA_034640	CADAFLAP00002839	Aminopeptidase Y, putative
AFLA_034870	CADAFLAP00002862	Putative uncharacterized protein
AFLA_035510	CADAFLAP00002926	RNA binding effector protein Scp160, putative
AFLA_035540	CADAFLAP00002929	Signal transducing adapter molecule, putative
AFLA_035620	CADAFLAP00002937	Hsp70 chaperone BiP/Kar2, putative
AFLA_036070	CADAFLAP00002982	Maleylacetoacetate isomerase MaiA
AFLA_036110	CADAFLAP00002986	4-hydroxyphenylpyruvate dioxygenase, putative
AFLA_036440	CADAFLAP00003019	Alanine aminotransferase, putative
AFLA_036640	CADAFLAP00003039	PH domain protein
AFLA_036840	CADAFLAP00003058	6-phosphogluconate dehydrogenase, decarboxylating
AFLA_037490	HCR1	Eukaryotic translation initiation factor 3 subunit EifCj, putative
AFLA_037960	CADAFLAP00003169	Glucosamine-fructose-6-phosphate aminotransferase
AFLA_038530	CADAFLAP00005181	Elastinolytic metalloproteinase Mep
AFLA_038700	CADAFLAP00005198	Putative uncharacterized protein
AFLA_038790	CADAFLAP00005207	Adenosine deaminase family protein
AFLA_039410	CADAFLAP00005268	Antigenic cell wall protein MP1
AFLA_040120	CADAFLAP00005339	Flavohemoprotein
AFLA_041260	CADAFLAP00005453	Ribosome associated DnaJ chaperone Zuotin, putative
AFLA_042090	CADAFLAP00005536	Fasciclin domain family protein
AFLA_042700	CADAFLAP00005597	Pre-mRNA splicing factor (Prp24), putative
AFLA_043390	CADAFLAP00005666	Hsp70 chaperone (BiP), putative
AFLA_043730	CADAFLAP00005700	Nuclear protein export protein Yrb2, putative
AFLA_044090	CADAFLAP00005736	Uricase (EC 1.7.3.3)(Urate oxidase)
AFLA_044620	CADAFLAP00005789	Mitochondrial Hsp70 chaperone (Ssc70), putative
AFLA_044820	CADAFLAP00005809	Glucose-6-phosphate isomerase (EC 5.3.1.9)
AFLA_045330	CADAFLAP00005860	Eukaryotic translation initiation factor subunit eIF-4F, putative
AFLA_045750	CADAFLAP00005902	Antigenic mitochondrial protein HSP60, putative
AFLA_045980	dpp5	Secreted dipeptidyl peptidase DppV
AFLA_046730	CADAFLAP00006000	Putative uncharacterized protein
AFLA_047350	pepP	Prolidase pepP, putative
AFLA_048510	CADAFLAP00010843	UV excision repair protein (RadW), putative
AFLA_048610	CADAFLAP00010853	Succinyl-CoA synthetase alpha subunit, putative

Table S6. Cont.

ID	STRING ID	Annotation
AFLA_050690	CADAFLAP00011061	Mitochondrial ADP,ATP carrier protein (Ant), putative
AFLA_051140	bglA	Beta-glucosidase, putative
AFLA_051770	CADAFLAP00011168	Thioredoxin reductase (EC 1.8.1.9)
AFLA_052400	CADAFLAP00011231	Isocitrate lyase AcuD
AFLA_054750	CADAFLAP00006084	Malate dehydrogenase, NAD-dependent
AFLA_055060	CADAFLAP00006115	NAD-dependent formate dehydrogenase AciA/Fdh
AFLA_055230	CADAFLAP00006132	Actin Act1
AFLA_055450	CADAFLAP00006154	Translation elongation factor eEF-1 subunit gamma, putative
AFLA_056170	CADAFLAP00006226	Catalase (EC 1.11.1.6)
AFLA_056260	CADAFLAP00006235	Nascent polypeptide-associated complex (NAC) subunit, putative
AFLA_056350	CADAFLAP00006244	2-methylcitrate dehydratase, putative
AFLA_056850	CADAFLAP00006294	Electron transfer flavoprotein, beta subunit, putative
AFLA_057240	CADAFLAP00006333	RNP domain protein
AFLA_057670	CADAFLAP00006376	Neutral protease 2, putative
AFLA_057770	plyA	Pectate lyase A
AFLA_058390	NTH1	DNA repair protein Ntg1, putative
AFLA_060110	CADAFLAP00006620	Putative uncharacterized protein
AFLA_060260	CADAFLAP00006635	Heat shock protein Hsp30/Hsp42, putative
AFLA_061880	CADAFLAP00006797	Pyridoxine biosynthesis protein
AFLA_066270	CADAFLAP00008051	RNA-binding La domain protein
AFLA_067940	CADAFLAP00008218	Putative uncharacterized protein
AFLA_068840	CADAFLAP00008308	Glycine cleavage system T protein
AFLA_069010	CADAFLAP00008325	S-adenosylmethionine synthetase (EC 2.5.1.6)
AFLA_069370	CADAFLAP00008361	Phosphoglycerate kinase (EC 2.7.2.3)
AFLA_069590	CADAFLAP00008383	Adenosylhomocysteinase (EC 3.3.1.1)
AFLA_070490	CADAFLAP00008473	Putative uncharacterized protein
AFLA_070990	CADAFLAP00008523	UTP-glucose-1-phosphate uridylyltransferase Ugp1, putative
AFLA_071010	CADAFLAP00008525	Heat shock protein (Sti1), putative
AFLA_073480	CADAFLAP00000131	Tropomyosin, putative
AFLA_074520	aglA	Alpha-galactosidase, putative
AFLA_076170	CADAFLAP00000399	Putative uncharacterized protein
AFLA_076180	CADAFLAP00000400	Putative uncharacterized protein
AFLA_076450	CADAFLAP00000427	Electron transfer flavoprotein alpha subunit, putative
AFLA_076710	CADAFLAP00000453	Malate dehydrogenase, NAD-dependent
AFLA_077860	CADAFLAP00000568	Putative uncharacterized protein
AFLA_078380	CADAFLAP00000620	Acetyl-coA hydrolase Ach1, putative
AFLA_078650	CADAFLAP00000647	ATP synthase subunit alpha
AFLA_078900	CADAFLAP00000672	Beta-N-acetylhexosaminidase NagA, putative
AFLA_079480	CADAFLAP00000730	Oligopeptidase family protein
AFLA_080390	CADAFLAP00000821	6-phosphogluconolactonase, putative
AFLA_080930	CADAFLAP00000874	EF hand domain protein
AFLA_082510	CADAFLAP00001031	TCTP family protein
AFLA_083370	CADAFLAP00001117	Glutathione oxidoreductase Glr1, putative
AFLA_086710	CADAFLAP00001450	Inorganic diphosphatase, putative
AFLA_086900	CADAFLAP00001469	Alpha-mannosidase

Table S6. Cont.

ID	STRING ID	Annotation
AFLA_087950	CADAFLAP00001573	Isocitrate dehydrogenase LysB
AFLA_088570	CADAFLAP00001635	Putative uncharacterized protein
AFLA_089270	CADAFLAP00008613	BZIP transcription factor HacA
AFLA_089380	CADAFLAP00008624	Extracellular chitosanase CsnC, putative
AFLA_090690	CADAFLAP00008754	Mycelial catalase Cat1
AFLA_091030	CADAFLAP00008788	Putative uncharacterized protein
AFLA_091060	CADAFLAP00008791	Allergen Asp F3
AFLA_091270	CADAFLAP00008812	Fumarate hydratase, putative
AFLA_091990	CADAFLAP00008884	Peptidyl-prolyl cis-trans isomerase (EC 5.2.1.8)
AFLA_092640	CADAFLAP00008949	Alkaline phosphatase (EC 3.1.3.1)
AFLA_093220	CADAFLAP00009007	Ran-specific GTPase-activating protein 1, putative
AFLA_093280	CADAFLAP00009013	Disulfide isomerase (TigA), putative
AFLA_093660	CADAFLAP00009051	Solid-state culture expressed protein (Aos23), putative
AFLA_094630	CADAFLAP00009148	Triosephosphate isomerase (EC 5.3.1.1)
AFLA_095570	CADAFLAP00009242	RNA annealing protein Yra1, putative
AFLA_095660	CADAFLAP00009251	Immunoglobulin G-binding protein H, putative
AFLA_097190	CADAFLAP00011457	Putative uncharacterized protein
AFLA_097750	CADAFLAP00011513	Putative uncharacterized protein
AFLA_099000	CADAFLAP00011638	Superoxide dismutase [Cu-Zn] (EC 1.15.1.1)
AFLA_099220	CADAFLAP00011660	Putative uncharacterized protein
AFLA_099650	CADAFLAP00011702	Woronin body major protein, putative
AFLA_101160	CADAFLAP00011853	40S ribosomal protein S9
AFLA_101930	CADAFLAP00011930	Succinate-semialdehyde dehydrogenase, putative
AFLA_102010	CADAFLAP00011938	Class V chitinase, putative
AFLA_103940	CADAFLAP00012129	Glutamate carboxypeptidase, putative
AFLA_105920	pgaB	Polygalacturonase A Precursor (EC 3.2.1.15)
AFLA_106310	CADAFLAP00004367	Chaperonin, putative
AFLA_106350	CADAFLAP00004371	ATP citrate lyase subunit (Acl), putatibe
AFLA_108100	CADAFLAP00004546	Argininosuccinate synthase (EC 6.3.4.5)
AFLA_108410	CADAFLAP00004576	Dipeptidase, putative
AFLA_108790	CADAFLAP00004614	Aldehyde dehydrogenase AldA, putative
AFLA_109320	CADAFLAP00004666	3-hydroxyisobutyrate dehydrogenase
AFLA_110160	dpp4	Extracellular dipeptidyl-peptidase Dpp4
AFLA_110260	CADAFLAP00004760	Flavin-binding monooxygenase-like protein
AFLA_110600	CADAFLAP00004794	Aminopeptidase
AFLA_110690	CADAFLAP00004803	Cytochrome c peroxidase Ccp1, putative
AFLA_111100	CADAFLAP00004844	Mitochondrial peroxiredoxin Prx1, putative
AFLA_112120	CADAFLAP00004946	Pyruvate carboxylase, putative
AFLA_112130	CADAFLAP00004947	Clathrin heavy chain
AFLA_112470	CADAFLAP00004981	Cobalamin-independent methionine synthase MetH/D
AFLA_112910	CADAFLAP00005025	Putative uncharacterized protein
AFLA_113000	CADAFLAP00005034	Integral ER membrane protein Scs2, putative
AFLA_117760	CADAFLAP00012348	Phytase, putative
AFLA_117850	CADAFLAP00012357	RPEL repeat protein
AFLA_119040	CADAFLAP00012476	Muramidase, putative

Table S6. Cont.

ID	STRING ID	Annotation
AFLA_119660	CADAFLAP00012538	ATP synthase subunit beta (EC 3.6.3.14)
AFLA_120630	CADAFLAP00012635	Glyoxylate/hydroxypyruvate reductase, putative
AFLA_122110	CADAFLAP00012783	Bifunctional catalase-peroxidase Cat2
AFLA_122180	CADAFLAP00012790	Putative uncharacterized protein
AFLA_122720	CADAFLAP00012843	Actin binding protein, putative
AFLA_124380	CADAFLAP00009496	Putative uncharacterized protein
AFLA_124500	CADAFLAP00009508	Nitric oxide synthase, putative
AFLA_125270	CADAFLAP00009585	Tyrosinase
AFLA_126870	CADAFLAP00009745	Putative uncharacterized protein
AFLA_127390	CADAFLAP00009797	Proteasome regulatory particle subunit Rpt5, putative
AFLA_128280	CADAFLAP00009886	M protein repeat protein
AFLA_128530	CADAFLAP00009911	Delta-1-pyrroline-5-carboxylate dehydrogenase PrnC
AFLA_130150	CADAFLAP00010073	NAD+ dependent glutamate dehydrogenase, putative
AFLA_130310	CADAFLAP00010089	Protein disulfide isomerase Pdi1, putative
AFLA_131280	CADAFLAP00007007	Actin cortical patch assembly protein Pan1, putative
AFLA_132540	CADAFLAP00007133	Thioredoxin TrxA
AFLA_133150	CADAFLAP00007194	Catechol dioxygenase, putative
AFLA_133920	CADAFLAP00007271	Acyl CoA binding protein family
AFLA_133990	CADAFLAP00007278	Secretory pathway gdp dissociation inhibitor
AFLA_134120	CADAFLAP00007291	Orotate phosphoribosyltransferase
AFLA_134340	CADAFLAP00007312	BAR domain protein
AFLA_135200	CADAFLAP00007398	Putative uncharacterized protein
AFLA_135540	CADAFLAP00007432	ATP synthase
AFLA_136570	CADAFLAP00007535	Cytochrome c
AFLA_136640	CADAFLAP00007542	Translation elongation factor EF-2 subunit, putative
AFLA_139300	CADAFLAP00007806	Ver-1
AFLA_139470	cpaO	FAD dependent oxidoreductase, putative
AFLA_139480	dmaT	Dimethylallyl tryptophan synthase, putative
